# PM_2.5_ induced neurotoxicity and depression unveiling exposure risks and mechanistic insights

**DOI:** 10.1016/j.isci.2026.116617

**Published:** 2026-07-17

**Authors:** Zonghao Ma, Suk-yu Yau

**Affiliations:** 1 Department of Rehabilitation Sciences, Hong Kong Polytechnic University, Hung Hom, Hong Kong SAR, China; 2 Research Institute of Smart Ageing, Hong Kong Polytechnic University, Hung Hom, Hong Kong SAR, China

**Keywords:** PM_2.5_, depression-like behavior, neurotoxicity

## Abstract

Fine particulate matter PM_2.5_ is increasingly linked to neuropsychiatric burden beyond its established cardiopulmonary effects. This narrative review integrates epidemiological, animal, and mechanistic evidence connecting PM_2.5_ exposure with depression and depression-related phenotypes. Human studies across different populations generally associate long-term PM_2.5_ exposure with elevated depression risk or symptom severity, with vulnerable windows including prenatal development and late life. Animal studies support biological plausibility by showing depression-like behaviors, electrophysiological changes, and neural injury after PM_2.5_ exposure. Component-focused evidence implicates metals, black carbon, organic compounds, and other constituents in oxidative stress, neuroinflammation, blood-brain barrier disruption, synaptic dysfunction, HPA axis dysregulation, neurotransmitter imbalance, and alterations in the kynurenine pathway. These findings support PM_2.5_ as a potential environmental risk factor for depression and highlight the need for component-resolved exposure assessment and mechanism-guided prevention strategies.

## Graphical abstract

**Figure:**
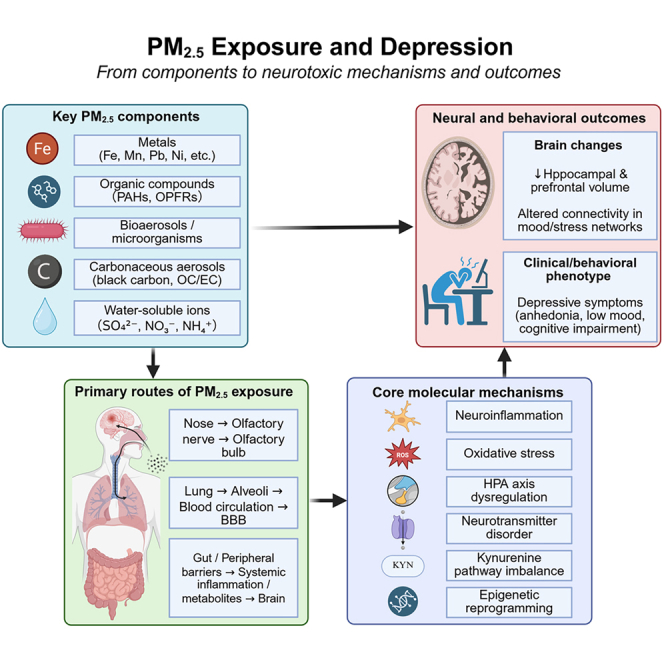

## Teaser

environmental science; environmental health; pollution

## Introduction

**Figure 1:**
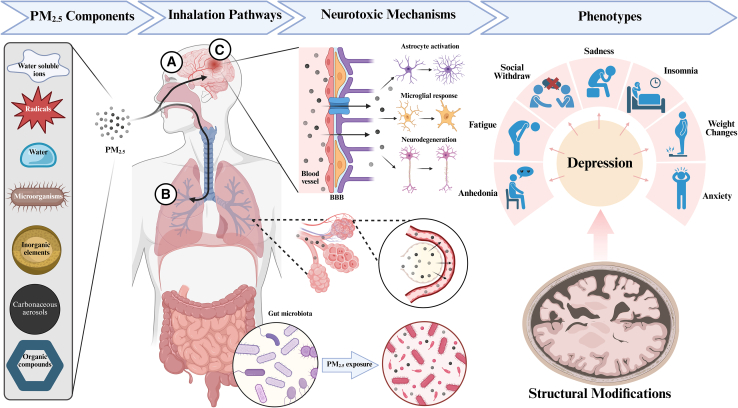
A schematic diagram of proposed mechanisms by which PM_2.5_ induces depression PM_2.5_, a complex mixture of water-soluble ions, inorganic elements, carbonaceous aerosols, free radicals, organic compounds, microorganisms, and adsorbed water, gains access to the brain through three primary routes: (A) olfactory translocation, where nanoparticles travel through olfactory nerves to the olfactory bulb; (B) systemic circulation, which causes body-wide inflammation that weakens the BBB; and (C) direct infiltration of ultrafine particles through the BBB, disrupting neurovascular function. After penetrating into the brain tissue, PM_2.5_ activates astrocytes and microglia, driving the release of pro-inflammatory cytokines and reactive oxidative stress (ROS). This neuroinflammatory cascade disrupts synaptic plasticity, reduces hippocampal neurogenesis, and induces oxidative damage to neurons and glia. Additionally, PM_2.5_ can induce systemic inflammation and changes in gut microbiota. Cumulatively, these molecular and cellular alterations promote structural remodeling in emotion-regulating brain regions, culminating in depressive symptomatology. Figure created with BioRender.

Particulate matter ≤2.5 μm in diameter (PM_2.5_), a ubiquitous air pollutant, has emerged as a critical environmental determinant of the global disease burden. Characterized by its aerodynamic diameter and complex physicochemical composition as detailed in Figure 1; PM_2.5_ penetrates deep into pulmonary alveoli and translocates systemically, posing multisystem health risks.^1^^,^^2^ While cardiopulmonary morbidity has dominated PM_2.5_ research, accumulating epidemiological and experimental evidence now implicates PM_2.5_ as a neurotoxicant capable of inducing neuropsychiatric disorders, particularly major depressive disorder (MDD).^3^^,^^4^ Alarmingly, longitudinal studies associate chronic PM_2.5_ exposure with elevated depression incidence and symptom severity, disproportionately affecting vulnerable populations in urbanized or industrialized regions.^5^^,^^6^^,^^7^

PM_2.5_ can invade the brain via three primary routes, underpinning its neurotoxic potential: (A) olfactory translocation, where nanoparticles travel along the olfactory nerve to the olfactory bulb; (B) systemic circulation, which promotes peripheral inflammation and blood-brain barrier (BBB) disruption; and (C) direct translocation of ultrafine particles across the BBB, leading to neurovascular impairment.^8^ Upon reaching the brain, PM_2.5_ components activate glial cells, disrupt redox equilibrium, and impair synaptic homeostasis, processes central to depression pathophysiology.^4^ Additionally, PM_2.5_ can induce systemic inflammation after penetrating the alveoli blood barrier and changes in gut microbiota to further activate the HPA axis, which are closely related to depression.^9^^,^^10^ Notably, the heterogeneous composition of PM_2.5_ dictates divergent neurotoxic outcomes, for example, transition metals like iron catalyze reactive oxygen species (ROS), organic constituents dysregulate mitochondrial function, and adsorbed pollutants synergistically amplify neuroinflammation.^11^ These multiscale insults could converge to remodel emotion-processing brain regions, including the frontal cortex, hippocampus, and amygdala, fostering depressive phenotypes.^8^

Despite growing epidemiological evidence showing the linkage between PM_2.5_ exposure and depression, critical knowledge about the underlying mechanisms is still largely unknown. Furthermore, PM_2.5_ component-specific neurotoxicity is poorly characterized. Whether metals, organics, or microbial constituents like bacterial endotoxins or fungal spores contributing to depressive outcomes has yet to be explored. A systems-level framework integrating PM_2.5_’s physicochemical properties, spatiotemporal exposure patterns, and multi-level neural perturbations, spanning molecular pathways, cellular dysfunction, and brain network alterations, is urgently needed to inform prevention and intervention strategies.^12^

**Figure 2:**
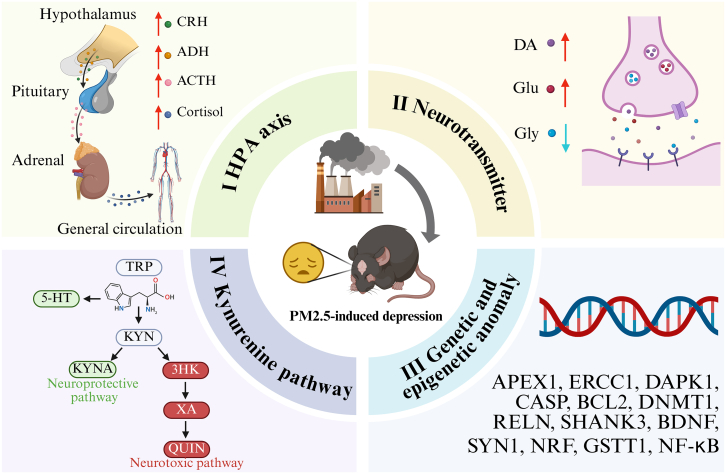
Proposed potential mechanisms underlying the molecular pathogenesis of PM_2.5_-induced depression (I) HPA axis dysfunction: PM_2.5_ exposure disrupts HPA axis regulation through inflammatory cytokines, elevating corticotropin-releasing hormone (CRH), vasopressin (ADH), adrenocorticotropic hormone (ACTH), and cortisol levels; (II) neurotransmitter hypothesis: PM_2.5_ exposure disrupts neurotransmitter homeostasis like dopamine activation, glycine suppression, and glutamate excitotoxicity; (III) genetic and epigenetic anomaly hypothesis: some genes are susceptible in PM_2.5_-induced depression, including DNA repair genes (APEX1 and ERCC1), apoptosis genes (DAPK1, CASP, and BCL2), epigenetic regulation gene (DNMT1), neurodevelopment genes (RELN, SHANK3, BDNF, and SYN1), oxidative stress-related genes (NRF and GSTT1), and inflammation gene (NF-κB); (IV) kynurenine pathway hypothesis: growing research indicates that PM_2.5_ exposure disrupts the kynurenine pathway, a key regulator of inflammatory and neuroimmune interactions. Figure created with BioRender. Apurinic/apyrimidinic endodeoxyribonuclease 1 (APEX1), excision repair cross-complementation group 1 (ERCC1), death-associated protein kinase 1 (DAPK1), caspase (CASP), B cell lymphoma 2 (BCL2), DNA (cytosine-5)-methyltransferase 1 (DNMT1), reelin (RELN), SH3 and multiple ankyrin repeat domains protein 3 (SHANK3), brain-derived neurotrophic factor (BDNF), synapsin I (SYN1), nuclear factor erythroid 2-related factor (NRF), glutathione S-transferase theta 1 (GSTT1), nuclear factor kappa-light-chain-enhancer of activated B cells (NF-κB).

This narrative review summarizes translational evidence linking PM_2.5_ exposure to depression risk and its potential underlying mechanisms. We critically evaluate (1) epidemiological evidence and potential PM_2.5_ neurotoxic components; (2) effects of PM_2.5_-exposed animal models on inducing depression-like behaviors; and (3) its related neural mechanisms of PM_2.5_-induced brain structural, cellular, and molecular changes, including PM_2.5_-driven neuroinflammation, HPA axis dysfunction, neurotransmitter dysfunction, genetic and epigenetic anomaly, and the kynurenine pathway (Figure 2). PM_2.5_ may directly translocate to the brain; however, majority of inhaled PM_2.5_ could initially interact with peripheral barrier systems, including the respiratory mucosa, gastrointestinal epithelium, skin, and ocular surface. Disruption of these interfaces may trigger chronic inflammation, immune dysregulation, and metabolic and neuroendocrine perturbations that could secondarily affect brain function via lung-brain, gut-brain, and skin-brain axes. These indirect pathways may represent the dominant routes by which environmental PM_2.5_ exposures influence depression risk in real-world settings.

This review provides a comprehensive summary of the existing findings, presenting the latest epidemiological observation identifying the association between PM_2.5_ and depression, the potential causal effects from animal models, and mechanistic insights from cellular/molecular studies. Moreover, we aimed to summarize the possible direct routes, including the lung, gut, skin, and ocular surface, in which PM_2.5_ triggers chronic inflammation, oxidative stress, and HPA-axis activation, and its indirect routes, including disruption of blood-brain barrier integrity, neuroinflammatory effects, and impairment in neural circuits, and synaptic plasticity in mood-regulating regions. Finally, we emphasize heterogeneity and vulnerability to specific PM_2.5_ components (metals, black carbon, organics related to depression neuropathology, vulnerability with exposure timing (prenatal, adolescence, late-life), and host factors (sex, genetic risk) that can shape depression risk.

## Evidence showing a linkage between PM_2.5_ exposure and risk of depression

Among youths, adults, and the elderly, Table 1 summarizes clinical evidence from Asia, Europe, and America consistently linking PM_2.5_ exposure to elevated MDD risk. In a south London cohort of 13,887 individuals aged ≥15 with psychotic or mood disorders, Newbury et al. identified residential air pollution exposure in correlation with heightened mental health service utilization, which is an indicator of illness severity and relapse. A rise in PM_2.5_ concentrations across interquartile ranges (IQRs) elevated the risk of in-patient days by 11% (95% CI, 3%–19%) and community mental health service events by 7% (95% CI, 4%–11%) after one year. These associations remained significant at the 7-year follow-up. Supporting this link, a meta-analysis by Braithwaite et al. confirmed a positive association between long-term PM2.5 exposure and depression risk, and also reported a potential connection with anxiety. Additionally, Kim et al. found that long-term exposure to PM_2.5_ elevates the risk of MDD in the general population, with a heightened susceptibility observed in individuals with chronic diseases among 27270 participants aged 15–79 years old in Seoul, Korea. Per 10 μg/m^3^ rise in PM_2_._5_, MDD hazard ratios during 2008–2010 follow-up were 1.44 (2007 levels; 95% CI, 1.17–1.78), 1.59 (2007–2010 cumulative; 95% CI, 1.02–2.49), and 1.47 (12-month moving average to event/censor; 95% CI, 1.14–1.90).

**Table 1:** Impact of PM_2.5_ exposure on MDD risk in clinical research

Age (years)	Duration	Locations	Exposure timing	Sample number	Key findings	References
9–11	2016–2021	USA	Exposure during ages 9–10	10783	1. > EPA’s 35 μg/m^3^ PM_2_._5_ standard → increased internalizing symptoms at baseline and one year. 2. Higher annual average PM_2_._5_ was weakly associated with higher baseline externalizing symptoms in females only.	Smolker et al.^13^
13–24	1991–2024	UK	1. The entire pregnancy period 2. Childhood: 0–9 years 3. Adolescence: 10–12 years	9065	1. Association between prenatal PM_2.5_ exposure and an elevated risk of depression (AOR = 1.10, *p* = 0.01). 2. Higher noise pollution exposure in childhood and adolescence was associated with increased odds of anxiety. 3. Pregnancy PM_2_._5_ exposure had a particularly strong association with later depression risk compared with exposure in later childhood.	Newbury et al.^14^
56.7 ± 8.1	2006–2010	UK	Long-term exposure during adulthood	389185	1. Long-term PM_2.5_ exposure increased depression risk (HR 1.15, *p* < 0.001). 2. Nonlinear PM_2.5_-depression relationship: steeper risk rise at low concentrations. 3. A stronger PM_2.5_-anxiety association in males (HR 1.18 vs. 1.07 in females). 4. Increased depression/anxiety risk with joint air pollution exposure (PM_2.5_, NO_2_, NO) raises (HR 1.16 and 1.11).	Yang et al.^15^
30.05 ± 5.81	2008–2016	Southern California, USA	Entire pregnancy and postpartum (0–6 months)	340679	1. O_3_ exposure (per IQR increase) linked to 9% higher postpartum depression (PPD) risk (AOR 1.09). 2. PM_10_ and PM_2.5_ exposure (per IQR) associated with 2% increased PPD risk. 3. PM_2.5_ organic matter and black carbon components significantly elevated PPD risk. 4. Critical exposure windows: O_3_ (entire pregnancy + postpartum), PM (third trimester + postpartum). 5. NO_2_ exposure showed no association with PPD. 6. Higher vulnerability in African American/Hispanic, educated, and underweight subgroups.	Sun et al.^16^
73.7 ± 4.8	2005–2016	USA	Long-term exposure during adulthood	8907422	1. Long-term PM_2_._5_ exposure (5 μg/m^3^ increase) linked to 0.91% higher depression risk. 2. Ozone (5 ppb increase) showed the strongest effect (2.13% risk increase). 3. Socioeconomically disadvantaged and comorbid patients are more vulnerable. 4. Sex/racial disparities: higher NO_2_ risk in men; higher PM_2_._5_ risk in black individuals.	Qiu et al.^6^
37–73	2006–2010	UK	Long-term exposure during adulthood	398241	1. Long-term PM_2_._5_ exposure linked to increased depression risk (OR = 1.08). 2. PM_10_, NO_2_, NO_x_, and PM_2_._5_ associated with incident mental disorders (HRs: 1.03–1.06). 3. The highest air pollution index quintile raises mental disorder risk by 11% (HR = 1.11). 4. Genetic predisposition enhances air pollution effects (OR = 1.20 in high-risk groups).	Gao et al.^7^
81.6 ± 3.6	2008–2018	USA	Long-term exposure in late life	764	1. Long-term PM_2_._5_/NO_2_ exposure accelerates depressive symptoms in older women. 2. Prefrontal cortex atrophy mediates 9%–13% of PM_2_._5_/NO_2_ effects on symptom progression. 3. Insula atrophy mediates 13% of NO_2_ effect.	Petkus et al.^17^
10 and 18	1994–2020	England and Wales	Childhood (age 10) and adolescence (age 18)	2039	1. The highest quartile of PM_2_._5_ exposure increases general psychopathology. 2. PM_2_._5_ linked to externalizing and thought disorder symptoms. 3. PM_2_._5_ effects overshadowed by NO_x_ in co-pollutant models.	Reuben et al.^18^
≥15	2008–2012	South London	3-month period around the first diagnosis	13887	1. PM_2_._5_ exposure linked to 11% increased in-patient days risk (1-year follow-up). 2. PM_2_._5_ exposure linked to 7% increased community mental health service events risk (1-year follow-up). 3. Dose-response relationship observed for PM_2_._5_ and service use.	Newbury et al.^19^
≥18	1974–2017	Around the world	Long-term or short-term exposure of the adult population (≥18 years old)	Study-dependent	1. Long-term PM_2_._5_ exposure increases depression risk (OR = 1.102 per 10 μg/m^3^). 2. Long-term PM_2_._5_ exposure shows positive associations with anxiety. Short-term PM_10_ exposure (0–2 days lag) elevates suicide risk.	Braithwaite et al.^20^
15–79	2007–2010	Seoul, Korea	Long-term exposure during adulthood	27270	1. Long-term PM_2_._5_ exposure (10 μg/m^3^ increase) increased MDD risk (HR = 1.44–1.59). 2. Higher MDD risk in individuals with underlying chronic diseases (diabetes: HR = 1.83; CVD: HR = 1.58; COPD: HR = 1.64). 3. Linear positive association between PM_2_._5_ levels (>26.7 μg/m^3^) and MDD.	Kim et al.^21^

Environmental Protection Agency (EPA), major depressive disorder (MDD), adjusted odds ratio (AOR), nitrogen dioxide (NO_2_), nitric oxide (NO), ozone (O_3_), postpartum depression (PPD), and particulate matter (PM).

PM_2.5_ exposure is associated with a higher likelihood of depression in pediatric populations, including 10783 children aged 9–11 years in the US.^13^ Childhood exposure to PM_2.5_ was correlated with heightened depression and anxiety symptoms at exposure and one-year post-exposure. The duration of exposure above US EPA standards appears to have a more significant effect on youth internalizing symptoms compared to annual averages or maximum daily exposure. Likewise, Newbury et al. affirmed that exposure to PM_2.5_ could increase the risk of MDD in youths. Their study involved 9,065 youths aged 13–24 years in the United Kingdom. An IQR rise of 0.72 μg/m^3^ in PM_2_._5_ exposure during pregnancy (AOR = 1.11; 95% CI, 1.04–1.19; *p* = 0.002) and childhood (AOR = 1.09; 95% CI, 1.00–1.10; *p* = 0.04) was associated with elevated odds of psychotic experiences. Prenatal PM_2_._5_ exposure was associated with depression (AOR 1.10; 95% CI, 1.02–1.18; *p* = 0.01), indicating that early-life air pollution contributes to depressive symptoms from adolescence through young adulthood. In addition, Reuben et al. reported that a specific PM_2_._5_ constituent was linked to elevated MDD risk among 18-year-olds in England and Wales. NOx exposure exhibited graded associations across secondary outcomes: weakest for internalizing symptoms (adj. *b* = 1.07, 95% CI 0.10–2.04, *p* = 0.03), moderate for externalizing (adj. *b* = 1.42, 95% CI 0.53–2.31, *p* = 0.002), and strongest for thought disorder (adj. *b* = 1.54, 95% CI 0.50–2.57, *p* = 0.004). These findings indicate that young individuals exposed to elevated outdoor NOx levels exhibited more severe psychopathology as they transitioned into adulthood.

In adults and the elderly, Yang et al. reported elevated MDD risk associated with PM_2_._5_ exposure in the study with 389,185 participants aged around 56.7 in the UK. Per IQR increase in PM_2_._5_, hazard ratios for depression and anxiety were 1.16 (95% CI, 1.09–1.23; *p* < 0.001) and 1.11 (95% CI, 1.05–1.17; *p* < 0.001), respectively. These elevated risks indicate sustained associations between higher PM_2_._5_ exposure and both outcomes across baseline and follow-up periods. Sun et al. identified sustained PM_2_._5_ exposure throughout the perinatal period as a significant risk factor for PPD, with 25,674 out of 340,679 participants (7.54%) having PPD in Southern California, USA. PM_2_._5_ constituents, particularly organic matter (OM) and black carbon, emerged as key PPD risk factors, alongside dose-dependent effects of overall PM_2_._5_ exposure during antepartum/postpartum periods (AOR 1.02 per IQR; 95% CI 1.00–1.03). Qiu et al. identified a significant correlation between chronic air pollution exposure and the likelihood of late-life depression diagnosis among 8,907,422 Medicare enrollers at the age of around 73.7 years old. Each 5-unit rise in chronic PM_2_._5_ exposure corresponded to a 0.91% elevation in depression risk (95% CI, 0.02%–1.81%). Although the underlying mechanisms are still largely unknown, Petkus et al. reported that reduced prefrontal cortex and insula volumes in community-dwelling older women may contribute to the rise in depressive symptoms linked to late-life PM_2.5_ exposure. Higher PM_2.5_ levels, by each IQR, were tied to a 0.022 increase in annual depressive symptom scores (95% CI, 0.003–0.042). Reduction in volume of the prefrontal cortex associated with PM_2.5_ exposure explained 13% of the link between higher depressive symptoms and PM_2.5_ levels.^17^

Overall, epidemiologic studies conducted across diverse regions and age groups support a moderately consistent, dose-dependent association between long-term PM_2.5_ exposure and depression incidence or symptom severity. Strengths of this body of work include large, population-based cohorts, repeated exposure assessments, and convergence of effect directions despite heterogeneous designs and settings. However, most studies are observational and therefore cannot fully rule out residual confounding by socioeconomic factors, noise, green space, lifestyle, and co-pollutants, and exposure misclassification may bias risk estimates toward the null. In addition, heterogeneity in outcome definitions (self-reported symptoms vs. clinical diagnoses), exposure metrics (annual averages vs. short-term peaks), and limited information on specific PM_2.5_ components and critical exposure windows complicate causal interpretation. These limitations underscore the need for complementary experimental models that can establish causality, test sensitive periods, and probe underlying mechanisms, which we address in the following sections on animal studies and neural pathways.

## PM_2.5_-induced depression-like phenotypes in animal models

According to the DSM-5 and ICD-10, a depressive episode is characterized by a persistent sad, irritable, or empty mood and a loss of interest or pleasure in usual activities for at least two weeks. This is accompanied by additional symptoms such as difficulty concentrating, excessive guilt or feelings of worthlessness, a bleak outlook, suicidal thoughts, sleep disturbances, changes in appetite or weight, and fatigue.^22^ In rodent models of depression, behaviors that mirror human MDD include a lack of enjoyment, anxiety, a sense of helplessness, and neglect of self-care and energy levels.

Recent research indicates a potential connection between exposure to PM_2.5_ and the development of MDD. Animal studies have also shown that PM_2.5_ could have adverse effects on brain health, leading to depressive phenotypes similar to those seen in individuals with MDD (Table 2).

**Table 2:** Impacts of PM_2.5_ exposure on the development of depressive-like symptoms in animal models

Exposure method	Animals	Duration	Exposure concentration (per day)	Behavioral tasks	Behavioral findings	References
Whole-body inhalation	Male C57BL/6J mice (eight weeks old)	8 h per day for 4, 6, and 8 weeks	Control group: 0.3 mg/(m^3^h) PM_2.5_ group: 2.2 mg/(m^3^h)	OFT	↓Total distance traveled ↓Time in the center zone ↓Distance in the center zone ↓Entries to the center zone	Li et al.^23^
				EPM	↓Total distance traveled ↓Percentage of open-arm entry time	
				EEG	↓Alpha waves ↓Beta waves	
Whole-body inhalation	Male C57BL/6J mice (seven weeks old)	5 months	Control group: 57.8 μg/m^3^ PM_2.5_ group: 185.2 μg/m^3^	OFT	↓Total distance traveled ↓Time in the center zone	Ji et al.^24^
				EPM	↓Time in the open arms ↑Time in the closed arms	
				MBT	↑Marble buried	
				FST	↑Immobility time	
Whole-body inhalation	Pathogen-free male Sprague-Dawley rats aged six weeks	12-week exposure: 6 h daily, 6 days/week	Filtered air group (FA): 0 μg/m^3^ Unconcentrated air group (UA): 46.37 ± 27.66 μg/m^3^ Concentrated air group (CA): 305.58 ± 254.22 μg/m^3^	OFT	↓Overall distance traveled: UA, CA ↓The route in Central Square: CA ↓Time spent in Central Square: CA	Chu et al.^25^
				SPT	↓Sucrose preference: CA	
				NSFT	↑Latency to feeding: UA, CA ↓Food consumption: CA	
	Six-week-old male Nrf2^−/−^ KO and WT mice	Daily 6-h exposures, 6 days/week for 9 weeks	Filtered air group: 0 μg/m^3^ Unconcentrated air group (UA): 48.25 ± 24.36 μg/m^3^	OFT	↓Time spent in Central Square: Nrf2 KO	
				TST	↑Immobility times: Nrf2 KO	
Intratracheal instillation	Kunming maternal mice (8–9 weeks old)	Every 3 days from E0 to parturition	Control group: No treatment Mock-treated group: 30 μL 1×PBS Low-dosage group: 30 μL 0.2592 μg/μL Medium-dosage group: 30 μL 1.56695 μg/μL High-dosage group: 30 μL 3.456 μg/μL	OFT	↓Total distance by high dose ↓The distance in the central area by high-dose	Zhang et al.^26^
				TST	↑Immobility of time by medium and high dose	

Filtered air (FA), unconcentrated air (UA), concentrated air (CA), knockout (KO), wildtype (WT), open field test (OFT), elevated plus maze (EPM), electroencephalogram (EEG), marble burying test (MBT), forced swim test (FST), sucrose preference test (SPT), novelty suppressed feeding test (NSFT), tail suspension test (TST).

In a study by Li et al., exposure to postnatal PM_2.5_ (2.2 mg/m^3^h) 8 h per day for 4, 6, and 8 weeks in adult male C57BL/6J mice via whole-body inhalation reduced exploratory activity and increased anxiety-like behavior in both open field test and elevated plus-maze test. Electroencephalogram (EEG) assessment revealed reduced alpha and beta wave activity in PM_2.5_-exposed mice, indicating that exposure to PM_2.5_ may disrupt normal brain states related to relaxation and attentive processes since alpha waves are primarily associated with the brain’s relaxed and inactive states, while beta waves are linked to processes such as anticipation, anxiety, and focused attention. Ji et al. reported that chronic 5-month PM_2.5_ exposure induced anxiety- and depression-like behaviors as well as reduced exploratory behavior in male mice. Chu et al. further documented PM_2.5_-induced depression-like behaviors in both rats and mice. Specifically, after 12 weeks of exposure, rats exhibited reduced locomotor activity in unconcentrated air and concentrated air groups relative to filtered air controls, with the concentrated air group displaying the most pronounced reduction in locomotion and reduction in sucrose preference. This suggests that PM_2.5_ exposure may impair motor activity and induce anhedonia-like behavior. In terms of feeding behavior, UA and CA rats showed delayed feeding initiation, suggesting the PM_2.5_-induced depression-like behavior in rats. In mice, exposure to UA did not significantly affect behavior, but *Nrf2*^−/−^ mice exposed to unconcentrated air showed increased immobility in the tail suspension test compared to its counterpart exposed to filtered air, suggesting that genetic factors, such as the absence of Nrf2 (a key regulator of antioxidant responses), may exacerbate the neurotoxic effects of PM_2.5_ in stress-related behaviors.^25^ Zhang et al. found that exposing pregnant Kunming mice to varying levels of PM_2.5_ via intratracheal instillation resulted in depression-like behaviors in their offspring. Specifically, the study used three different PM_2.5_ concentrations: low-dose (30 μL of 0.2592 μg/μL), medium-dose (30 μL of 1.56695 μg/μL), and high-dose (30 μL of 3.456 μg/μL). The highest PM_2.5_ dose (3.456 μg/μL) during pregnancy led to significant motor function defects in the offspring, including reduced total distance and the distance in the central area, as assessed by the open field test. Additionally, offspring from medium- and high-dose groups displayed increased immobility duration in the tail suspension test, indicating the prenatal PM_2.5_ exposure induced depressive behaviors, while the low-dose group exhibited no behavioral changes. These results demonstrate a dose-response gradient linking gestational PM_2.5_ exposure to neurobehavioral deficits in offspring.^26^

Taken together, animal studies demonstrate that PM_2.5_ exposure is sufficient to induce core depression- and anxiety-like behaviors, with converging evidence for dose- and duration-dependent effects and modulation by genetic background. Strengths of this literature include tightly controlled exposure conditions, incorporation of both prenatal and adult exposures, and the use of multiple behavioral assays and electrophysiological readouts that map onto human symptom domains. At the same time, most studies employ relatively high PM_2.5_ concentrations or bolus intratracheal instillation that exceed typical ambient human exposures, are conducted predominantly in male rodents of a limited number of strains, and seldom model common human comorbidities such as cardiometabolic disease. Behavioral paradigms also capture only selected dimensions of human MDD and are sensitive to laboratory context. Thus, while these models provide important biological plausibility and help isolate specific pathways, caution is warranted when extrapolating quantitative risk estimates or sensitive developmental periods to humans. These constraints motivate a shift from whole-mixture exposures to component-specific and pathway-focused approaches, which we review in the next section.

## Neurotoxic impacts of specific PM_2.5_ components

While epidemiological and preclinical studies have established a robust association between PM_2.5_ exposure and depression risk, the heterogeneous physicochemical composition of PM_2.5_ complicates efforts to pinpoint its neurotoxic mechanisms. PM_2.5_ is not a singular entity but a complex mixture of diverse components, including inorganic minerals, water-soluble ions, carbonaceous matter, organic molecules, free radicals, water, and airborne microorganisms, each with distinct toxicological properties.^1^ Understanding component-specific neurotoxic effects is critical for elucidating how PM_2.5_ drives depressive pathophysiology, as individual constituents may act synergistically, antagonistically, or independently to disrupt neural homeostasis. Emerging evidence suggests that the combined effects of specific PM_2.5_ components may be particularly detrimental, with interactions between metals and organic compounds potentially amplifying neurotoxicity.^11^ Furthermore, identifying high-risk components could inform targeted mitigation strategies and regulatory policies. Recent advancements have shed light on specific neurotoxic components within PM_2.5_. Despite these insights, further investigation is needed to fully characterize the neurotoxicity of specific PM_2.5_ components and their interactions. The subsequent sections review the specific constituents of PM_2.5_, examine evidence connecting these constituents to depression, and explore potential neurotoxic mechanisms that may underlie this association. Table 3 summarizes the major PM_2.5_ component classes, their representative constituents and emission sources, and the main neurotoxic effects and mechanisms that have been implicated in depression-related pathology.

**Table 3:** Major PM_2.5_ components: their representative constituents, sources, and implicated neurotoxic mechanisms

Component type/Main source	Contents	Neurotoxic effects	Implicated mechanisms	References
Inorganic elements/Soil dust and industry	Cd, Pb, As, S, Si, K, Cl, Ca, Na, Fe, Al, Mg, Mn, Br, Ba, Sb, Ni, Cr, Pt, and Ti	Cognitive impairment, depressive and anxiety symptoms, AD-like neuropathology, olfactory and hippocampal dysfunction	ROS generation, microglial activation, BBB disruption, and endothelial damage; mitochondrial dysfunction and synaptic loss	Boman et al.^27^; Bozlaker et al.^28^; Manousakas et al.^29^; Wei et al.^30^; Sălcudean et al.^31^; Calderón-Garcidueñas et al.^32^; Song et al.^33^; Liu et al.^34^; Liu et al.^35^; Lubczyńska et al.^36^; Calderón-Garcidueñas and de la Monte^37^; Cheng et al.^38^; Maher^39^; Meng et al.^40^; Calderón-Garcidueñas et al.^41^; Calderón-Garcidueñas et al.^42^; Guarneros et al.^43^
Water-soluble ions/Secondary formation from gaseous precursors, sea spray, and industrial emissions	(NH_4_)_2_SO_4_, NH_4_NO_3_, NH_4_HSO_4_, H_2_SO_4_, HNO_3_, Na^+^, K^+^, Mg^2+^, Ca^2+^, and Cl^−^	Depressive symptoms, dementia risk, poorer neonatal neurobehavioral scores, and neurodevelopmental vulnerability	Oxidative stress and membrane damage; amyloid-β aggregation; mitochondrial DNA depletion and impaired neuronal energy metabolism; modulation of glutamate and monoamine pathways	Yin et al.^44^; Hu et al.^45^; Kaumbekova et al.^46^; Shi et al.^47^; Li et al.^48^; Nunez et al.^49^; Sukumaran et al.^50^; van Wijngaarden et al.^51^; Peng et al.^52^; Bobermin et al.^53^; Lu et al.^54^
Carbonaceous aerosols/Vehicle emissions, biomass burning, and industrial processes	Organic carbon, elemental carbon	Increased depressive symptoms, cognitive delay, dementia, and stroke; neuronal loss and white-matter damage	Deep lung and brain deposition; neuroinflammation; oxidative stress and ferroptosis; vascular dysfunction and impaired neurovascular coupling	Meng et al.^40^; Li et al.^48^; Lu et al.^54^; Feng et al.^55^; Zhu et al.^56^; Wang et al.^57^; Shen et al.^58^; Vanbrabant et al.^59^; Sunyer et al.^60^; Sun et al.^61^; Guo et al.^62^; Ljungman et al.^63^; Lin et al.^64^; Mortamais et al.^65^; Duchesne et al.^66^; Sunyer et al.^67^
Organic compounds/Partial combustion, thermal decomposition of fuels and biomass, petroleum, natural emission releases, atmospheric gas oxidation, and photochemical processes	Polycyclic aromatic hydrocarbons (PAHs), volatile organic compounds (VOCs), oxygenated volatile organic compounds (OVOCs), and secondary organic aerosol (SOA)	Neurodevelopmental deficits, depression- and anxiety-like behaviors, AD-like changes, increased vulnerability to mood and cognitive disorders	Activation of Aryl Hydrocarbon Receptor (AhR) and other xenobiotic receptors; oxidative stress and lipid peroxidation; mitochondrial dysfunction; DNA methylation and other epigenetic changes; impaired synaptic plasticity and dendritic growth; tau hyperphosphorylation; ferroptosis in neurons	Shi et al.^47^; Nunez et al.^49^; Guo et al.^62^; Xu et al.^68^; Song et al.^69^; Hertz-Picciotto et al.^70^; Sun et al.^71^; Ma et al.^72^; Hou et al.^73^; Wang et al.^74^; Newell et al.^75^
Radicals/Radical generation via HONO photolysis (from soil nitrites/microbial activity) and tropospheric oxidation of unsaturated organic compounds	OH radicals, QOOH intermediates, ROS	Amplifiers of the toxicity of co-emitted PM_2.5_ constituents; neuronal and glial injury	Direct oxidation of lipids, proteins, and DNA; activation of redox-sensitive inflammatory pathways; promotion of microglial and astrocytic activation; disruption of mitochondrial function	Block et al.^76^; Block et al.^77^; Crounse et al.^78^; Halliwell et al.^79^; Lin and Beal^80^; Morgan and Liu^81^; Pöschl and Shiraiwa^82^; Su et al.^83^; Oswald et al.^84^; Valkoet al.^85^
Water/Atmospheric moisture	H_2_O, cloud condensation nuclei (CCN), aerosols	Modulating hygroscopic growth, deposition pattern, and solubility of toxic PM_2.5_ constituents to the lung and brain	Altering particle size and lung deposition, dissolution, and bioavailability of metals; neuroinflammation and oxidative stress	Block and Calderón-Garcidueñas^76^; Swietlicki et al.^86^; Pöschl^87^; Costa and Dreher^88^; Calderón-Garcidueñaset al.^89^
Inhalable microorganisms/Rural and urban areas	Biological constituents include viable bioaerosols (pollen, fungal/bacterial spores, viruses, plant/animal detritus) and non-viable microbial remnants	Neuroinflammation, mood, and cognitive changes	Activation of innate immune receptors by endotoxins and microbial components; cytokine release and chronic low-grade inflammation; modulation of gut and respiratory microbiota affecting HPA axis and neurotransmission	Block and Calderón-Garcidueñas^76^; Akira et al.^90^; Beutler^91^; Budden et al.^92^; Cryan and Dinan^93^; Dantzer et al.^94^; Despres et al.^95^; Douwes et al.^96^; Fonken et al.^97^; Foster and McVey Neufeld^98^; Fröhlich-Nowoisky et al.^99^; Man et al.^100^; Thorne^101^

Sulfur (S), silicon (Si), potassium (K), chlorine (Cl), calcium (Ca), sodium (Na), iron (Fe), aluminum (Al), magnesium (Mg), manganese (Mn), barium (Ba), bromine (Br), antimony (Sb), nickel (Ni), and chromium (Cr), cadmium (Cd), lead (Pb), arsenic (As), antimony (Sb), inductively coupled plasma mass spectrometry (ICPMS), inductively coupled plasma optical emission spectrometry (ICP-OES), time-of-flight mass spectrometry (TOFMS), energy-dispersive X-ray fluorescence (EDXRF), X-ray diffraction (XRD), scanning electron microscopy with energy dispersive spectroscopy (SEM-EDS), and high-resolution continuous source graphite furnace atomic absorption spectrometry (HR-CSGF AAS), Alzheimer’s disease (AD).

### Evidence showing the linkage between specific PM_2.5_ components and depression

Emerging evidence highlights the differential roles of specific PM_2.5_ chemical components in modulating depression risk. Short-term exposure to OM, sulfate (SO_4_^2−^), and ammonium (NH_4_^+^) in PM_2.5_ has been significantly associated with increased depression-related outpatient visits, with heightened susceptibility observed in females and older adults.^102^ Notably, phenyl-containing compounds in PM_2_._5_ organic extracts, identified as competitive binders to dopamine receptor DRD1, disrupt dopamine signaling and induce depression-like behaviors in mice.^24^ Prenatal and postnatal exposure to PM_2_._5_ organic constituents and black carbon demonstrated significant associations with increased postpartum depression incidence, highlighting perinatal susceptibility.^16^ Combustion-derived ultrafine particulate matter (UFPM) and industrial nanoparticles (NPs) in PM_2.5_ contribute to depressive pathology by triggering oxidative stress, systemic inflammation, and neuroinflammation.^103^ Epidemiological evidence confirmed PM_2_._5_ and carbon monoxide as independent predictors of psychotic-feature major depression, yet paradoxically, reduced concentrations of BaP, SO_2_, and Cd exhibited comparable risk associations. Conversely, ozone (O_3_) exhibited a negative correlation with depression incidence.^104^ Proximity to manganese (Mn) emission sources, leading to elevated PM_2_._5_-Mn exposure, correlated with increased depression symptomatology and current anxiety, though not lifetime anxiety.^105^ Gender-specific effects were also evident, with carbon monoxide and humidity linked to female suicide attempts, while humidity alone influenced male suicide rates.^106^

Collectively, these studies underscore the complex interplay between specific PM_2.5_ components, such as OM, black carbon, UFPM, NPs, and heavy metals, and depression risk. Mechanistically, components like phenyl-containing compounds could disrupt neurotransmitter signaling, while others may drive oxidative stress and inflammation. Vulnerable subgroups, including females, the elderly, and perinatal populations, could exhibit heightened susceptibility. These findings emphasize the importance of regulating source-specific PM_2.5_ emissions and the potential linkage between specific PM_2.5_ components and depression.

### Inorganic components

The elemental profile of PM_2_._5_ comprises complex inorganic constituents: alkali/alkaline earth metals (K, Na, Ca, Mg), metalloids (Si, As), transition metals (Fe, Mn, Ni, Cr), heavy metals (Cd, Pb), halogens (Cl), and chalcogens (S), alongside aluminum (Al).^27^^,^^28^ Advanced analytical techniques have been critical in identifying these components and their neurotoxic potential.^29^ Emerging evidence links these inorganic constituents to neuroinflammatory and neurodegenerative pathways implicated in depression.

Manganese (Mn) and lead (Pb), in synergy with aluminum (Al), are key drivers of CCR5-mediated microglial activation and neuroinflammation in murine models.^30^ Chronic neuroinflammation, characterized by elevated pro-inflammatory cytokines such as IL-1β and COX-2, disrupts synaptic plasticity and monoaminergic neurotransmission, central to depression pathophysiology.^31^ Similarly, nickel (Ni) and chromium (Cr) in PM_2.5_ amplify neuroinflammatory responses by increasing IL-1β and COX-2 levels, potentially exacerbating depressive symptoms through BBB disruption and neuronal apoptosis.^32^^,^^33^ Lead ions (Pb^2+^) further induce neuronal apoptosis and synaptic damage, while aluminum ions (Al^3+^) paradoxically mitigate cytotoxicity by suppressing ROS, suggesting potential metal-specific effects in depression-related neurotoxicity.^34^^,^^35^

Iron (Fe), a major component of vehicle emissions, could impair mitochondrial function and elevate oxidative stress in the brain regions involved in mood regulation.^36^^,^^37^^,^^38^ Magnetite nanoparticles rich in Fe generate ROS, damaging organelles and promoting neurodegeneration. Those cellular changes could also be linked to depression via oxidative stress-induced neuronal dysfunction.^39^ Barium (Ba), a marker of brake wear, and oxidative potential metrics are associated with increased malondialdehyde (MDA), a biomarker of oxidative stress, during early gestation (10–17 weeks).^40^ This suggests that prenatal exposure to PM_2.5_ metals may prime oxidative stress pathways, disrupting neurodevelopment and increasing vulnerability to mood disorders later in life.

Emerging data also implicate rare earth elements such as lanthanum and cerium and metals from electronic waste in early Alzheimer’s and Parkinson’s-like pathology, including tau hyperphosphorylation and α-synuclein aggregation, which may predispose individuals to comorbid neurodegeneration and depression.^41^^,^^42^ Children exposed to PM_2.5_ exhibit olfactory deficits and elevated hair Mn levels, suggesting metal-driven sensory and cognitive impairments that may precede or exacerbate depressive phenotypes.^43^

In summary, the neurotoxic effects of PM_2.5_ inorganic components may intersect with depression through shared mechanisms, including neuroinflammation, oxidative stress, BBB disruption, and synaptic dysfunction. Metals such as Mn, Pb, Ni, and Fe activate microglia, elevate pro-inflammatory cytokines, and generate ROS, impairing neuronal resilience and monoaminergic signaling. Rare earth elements and catalytic metals could further propagate neurodegenerative pathways that overlap with mood disorder etiology. Children and urban populations are particularly vulnerable, with early-life exposure potentially programming long-term depressive risk. Future research should prioritize longitudinal studies to disentangle the temporal and dose-dependent relationships between specific PM_2.5_ metals and depression, while therapeutic strategies targeting metal detoxification or anti-inflammatory pathways may offer potential intervention opportunities.

### Water-soluble ions

PM_2_._5_-associated depression involves neurotoxic water-soluble inorganic ions (WSIIs), particularly SNA ions (SO_4_^2−^, NO_3_^−^, NH_4_^+^), which dominate the composition and could disrupt neurological functions linked to depressive disorders.^44^ Under ammonium-deficient conditions, SNA derivatives like ammonium sulfate ((NH_4_)_2_SO_4_) and ammonium nitrate (NH_4_NO_3_) undergo dissociation to liberate acidic species (H_2_SO_4_, HNO_3_), potentiating oxidative stress and neuroinflammation.^45^ For instance, molecular dynamics simulations reveal that sulfate- and nitrate-rich PM_2.5_ accelerates amyloid-beta aggregation, a process associated with Alzheimer’s disease but also relevant to depression through shared pathways of synaptic dysfunction and neuroinflammation.^46^ Similarly, long-term exposure to sulfate-rich PM_2.5_, particularly from fossil fuel combustion, correlates with increased dementia and Alzheimer’s disease incidence, with neuroinflammatory cascades potentially bridging these outcomes to depressive symptomatology.^47^^,^^48^

Nitrate (NO_3_^−^) demonstrates paradoxical effects. While ammonium nitrate exposure is associated with impaired cognitive function and Parkinson’s disease progression, which is often comorbid with depression.^49^^,^^50^ Secondary nitrate from traffic emissions paradoxically reduces neurodegenerative hospital admissions, suggesting complex interactions between nitrate chemistry, BBB permeability, and neurotransmitter disruption.^51^ Such findings align with depression models involving dysregulated glutamate and monoamine systems. Additionally, long-term exposure to PM_2.5_-associated nitrate reduces mitochondrial DNA abundance, and this depletion may impair neuronal energy metabolism and amplify depressive symptoms linked to adenosine triphosphate (ATP)-dependent neurotransmitter recycling.^52^

Ammonium (NH_4_^+^), though less studied, may indirectly contribute to neurotoxicity by stabilizing acidic aerosols, thereby amplifying oxidative damage in neurons.^53^ Prenatal exposure to ammonium-rich PM_2.5_ mixtures during critical developmental windows reduces neonatal neurobehavioral scores, hinting at early-life vulnerabilities that may predispose individuals to mood disorders later in life.^54^

In summary, the neurotoxic impacts of PM_2.5_ water-soluble ions, particularly SNA, are increasingly tied to depression through overlapping mechanisms: (1) oxidative stress from acidic aerosols damaging neuronal mitochondria; (2) neuroinflammation triggered by amyloidogenic or pro-inflammatory ions like sulfate; and (3) neurodevelopmental disruption during critical prenatal periods. While sulfate emerges as a major contributor to cognitive and mood disorders, nitrate’s dual role underscores the importance of source-specific analyses. Mitigating SNA emissions, especially from fossil fuels, could reduce the dual burden of neurodegenerative and mood disorders.

### Carbonaceous aerosols

Organic carbon and elemental carbon, the primary components of carbonaceous aerosols, account for roughly 30% of PM_2.5_ mass and are significant contributors to its neurotoxic effects.^55^ Elemental carbon, primarily derived from vehicle emissions,^56^ and black carbon (a subset of elemental carbon linked to traffic and fossil fuel combustion) have emerged as particularly potent neurotoxic agents.^57^ Notably, exposure to black carbon correlates with depressive symptoms in university students, with gender and educational attainment modulating this relationship.^58^ Black carbon particles accumulate disproportionately in memory-associated brain regions, including the hippocampus, prefrontal cortex, and thalamus, which are implicated in mood regulation and depression pathophysiology.^59^ These findings suggest that black carbon-induced structural and functional disruptions in these regions may exacerbate depressive disorders through impaired neuroplasticity and synaptic signaling.

Dementia incidence shows a robust correlation with prolonged exposure to PM_2.5_ constituents, especially black carbon and sulfate, where black carbon is implicated in neurotoxic pathways.^48^ Similarly, traffic-related elemental carbon has been linked to reduced cognitive development,^60^ a risk factor for depression.

Emerging evidence highlights black carbon’s outsized contribution to systemic and neuroinflammatory outcomes. For instance, black carbon accounted for 71% of PM_2.5_ mixture effects on preeclampsia-eclampsia risk,^61^ a condition tied to inflammatory dysregulation. Prenatal exposure to black carbon is also associated with oxidative stress biomarkers and neonatal neurobehavioral deficits,^40^^,^^54^ suggesting early-life neurodevelopmental vulnerabilities that may predispose individuals to depression later in life. Furthermore, black carbon-driven ferroptosis in neuronal cells^62^ aligns with depression-associated neuronal loss in mood-regulatory circuits. Paradoxically, while black carbon consistently correlates with dementia and stroke,^63^^,^^64^ some studies report null associations with dementia^65^ or white matter hyperintensities,^66^ highlighting the need for mechanistic clarity. Mortamais et al. reported no link between exposure to black carbon and dementia risk in the three-city study. However, Ljungman et al. found black carbon from traffic exhaust was associated with incident stroke. From the perspective of source specificity, Ljungman et al. highlighted traffic-derived black carbon as the primary driver of stroke risk, whereas Mortamais et al. did not differentiate black carbon sources. In terms of outcome heterogeneity, dementia involves long-term neurodegenerative processes, while stroke reflects acute cerebrovascular events. black carbon’s pro-thrombotic effects may disproportionately impact stroke risk.

Collectively, the neurotoxic impacts of PM_2.5_ carbonaceous aerosols, particularly black carbon and elemental carbon, converge on oxidative stress, neuroinflammation, and neuronal damage—processes central to depression pathogenesis. black carbon’s preferential accumulation in mood- and memory-related brain regions, combined with its role in ROS generation and ferroptosis, provides a possible biological link to depressive symptomology. While traffic-derived elemental carbon impairs cognitive and attentional function,^67^ black carbon’s contribution to prenatal oxidative stress and neurodevelopmental deficits may underscore early susceptibility to mood disorders. However, inconsistencies in epidemiological findings emphasize the importance of source specificity and outcome heterogeneity.

### Organic compounds

The organic constituents of PM_2.5_ play a critical role in mediating neurotoxic effects, with emerging evidence linking their mechanisms to depression-related pathways. Polycyclic aromatic hydrocarbons (PAHs), such as benzo(*a*)pyrene, induce oxidative stress, DNA methylation changes, and mitochondrial dysfunction, leading to neurodevelopmental abnormalities and neurodegenerative disorders like Alzheimer’s disease.^68^ These mechanisms overlap with depression pathophysiology, which is characterized by oxidative damage and mitochondrial impairment.^69^ Notably, prenatal PAH exposure alters immune and neurodevelopmental trajectories, potentially predisposing individuals to neurobehavioral disorders.^70^

Brominated flame retardants like decabromodiphenyl ether (BDE-209) disrupt synaptic plasticity, dendritic growth, and tau protein regulation.^71^^,^^72^ Such synaptic dysfunction and tauopathy mirror deficits observed in depression, where impaired neuronal communication and neurotrophic signaling are hallmarks. Prenatal PM_2.5_ organic components, particularly PAHs, impair spatial learning and memory in mice via disrupted Hoxa5-mediated neuronal morphogenesis,^73^ suggesting early-life exposure may prime vulnerability to mood disorders. Furthermore, PM_2.5_ organic extracts induce ferroptosis in neuronal cells,^62^ a form of iron-dependent cell death linked to neurodegeneration and depressive pathology through oxidative stress.

Organophosphate flame retardants (OPFRs) in PM_2.5_, particularly chlorinated variants from plastic waste, pose noncarcinogenic and carcinogenic risks.^74^ While direct evidence linking OPFRs to depression is limited, their neurotoxic effects on immune and endocrine systems may indirectly influence mood regulation.^75^ Similarly, particulate OM is associated with dementia and Parkinson’s disease progression,^47^^,^^49^ disorders often comorbid with depression, implying shared pathways such as neuroinflammation and oxidative stress.

Collectively, PM_2.5_ organic components, including PAHs, SOAs, OVOCs, BDE-209, and OM, exert neurotoxicity via oxidative stress, synaptic dysfunction, mitochondrial damage, and immune dysregulation, which could be potential mechanisms linked to depression etiology. Prenatal and long-term exposure to these compounds disrupts neurodevelopment, synaptic plasticity, and neurotrophic signaling, potentially increasing depression risk. However, critical gaps remain, particularly regarding the direct effects of OPFRs on mood-related pathways.

Across studies, several PM_2.5_ constituents, including transition and heavy metals (Fe, Mn, Pb), secondary inorganic ions, black and elemental carbon, and organic compounds such as PAHs and flame retardants, emerge as plausible drivers of neurotoxicity relevant to depression. Evidence comes from source-apportioned epidemiologic analyses, *in vivo* inhalation and instillation models, and *in vitro* systems demonstrating oxidative stress, neuroinflammation, synaptic dysfunction, and neurodegeneration. Nonetheless, component-specific conclusions remain tentative because constituents co-occur and are highly correlated in real-world mixtures, source profiles differ across regions, and incomplete chemical speciation limits exposure resolution. Experimental studies often test isolated components or enriched fractions at concentrations that may not faithfully reproduce realistic co-exposure scenarios. Future work integrating multi-pollutant statistical methods, source-resolved exposure assessment, and experimental mixture designs will be essential to identify the most neurotoxic components and sources. Importantly, the pathways implicated by these components converge on a relatively restricted set of neural mechanisms, including neuroinflammation, oxidative stress, BBB disruption, and neurotransmitter imbalance, which are discussed in detail in the subsequent mechanistic section.

## Neural mechanisms by which PM_2.5_ induces depression

### Brain volume changes

Chronic PM_2.5_ exposure is linked to structural brain deterioration and atrophy in regions governing emotion and stress processing, notably the prefrontal cortex (PFC) and hippocampus, as evidenced by consistent research.^107^^,^^108^ Elevated mean PM_2.5_ concentrations over 5–20 years predict volumetric reductions in deep-gray matter and diminished total brain volume.^109^ Prenatal exposure to PM_2.5_ also appears to have neurodevelopmental consequences, with studies linking it to decreased corpus callosum volumes and behavioral disorders in children,^110^ with a higher risk of hospitalization for depression observed at an older age.^111^ Even minimal PM_2.5_ exposure is associated with reduced cortical surface area and thickness in the frontal, parietal, temporal, occipital, and cingulate regions, with distinct patterns observed between the hemispheres.^4^^,^^112^ The hippocampus, crucial for emotional function, is particularly vulnerable, with PM_2.5_ exposure associated with pathological changes and reduced hippocampal volume.^113^ Additionally, cerebral cortical impairments, widely linked to depression pathophysiology, are observed following PM_2.5_ exposure.^25^^,^^114^

Significantly, reduced white matter volume was detected exclusively in women with lower adherence to a Mediterranean-Dietary Approaches to Stop Hypertension (DASH) diet intervention for neurodegenerative delay, indicating dietary patterns may mitigate air pollution’s neurotoxic effects.^115^ Additionally, smaller PFC and insula volumes may mediate increased depressive symptoms linked to late-life exposure to NO_2_ and PM_2.5_.^17^ Interestingly, Herting et al. (2024) proposed that air pollution does not affect global brain structure, such as overall brain size. However, Wilker et al. (2015) observed that exposure to high levels of PM_2.5_ air pollution is linked to reduced whole brain volume, a sign of accelerated brain aging, and an increased risk of silent brain infarcts, even in individuals without dementia or stroke. These subtle yet harmful effects on brain structure may also contribute to depression, as brain atrophy and vascular damage are known risk factors for mood disorders. Collectively, these findings underscore that PM_2.5_ does seem to have a more pronounced impact on specific brain regions critical for emotional regulation and stress response (Table 4). This localized effect may be particularly relevant to understanding the neurobiological underpinnings of depression.

**Table 4:** Summary of brain volume changes induced by PM_2.5_ exposure

Brain volume change	PM_2.5_ concentration	Exposure duration	Geographic coverage/Sample size	Age (year)/Sex	References
Left putamen ↓	1.72–15.9 μg/m^3^	∼1 year	21 US sites 10343	9–10.99 Males: 52.3% Females: 47.7%	Cserbik et al.^112^
Left pallidum ↓					
Corpus callosum ↓	Pregnancy median: 16.8 μg/m^3^ (IQR: 16.6–17.2) Childhood median: 16.7 μg/m^3^ (IQR: 16.5–17.0)	Pregnancy: ∼9 months from conception to birth Childhood: ∼9 years from birth to MRI scan	Netherlands, Rotterdam 3133	9–12 Males: 50% Females: 50%	Lubczyńska et al.^116^
Hippocampus ↓					
Whole brain volume No change					
Insula ↓	11.33 ± 2.46 μg/m^3^	3 years	US 764	81.6 ± 3.6 Females	Petkus et al.^17^
Prefrontal cortex ↓					
Deep-gray volume ↓	9.4–19.1 μg/m^3^	8 years 17 years	US Minnesota, Maryland, North Carolina, Mississippi 1753	76 Males: 40% Females: 60%	Power et al.^109^
Whole brain volume ↓	7.7–17.6 μg/m^3^	N/A	New England, USA 943	Median (IQR): 68 (9) Males: 48% Females: 52%	Wilker et al.^108^
Corpus callosum ↓	23.6 μg/m^3^	Prenatal period (entire pregnancy)	Barcelona, Spain 186	8–12 Males: 51% Females: 49%	Mortamais et al.^110^
White matter volume ↓	9.3–14.9 μg/m^3^	3 years	US 1302	Around 74–85 Females	Chen et al.^115^

Magnetic resonance imaging (MRI), interquartile range (IQR), not mentioned (N/A).

### BBB leakage

The BBB forms a selective semipermeable shield between blood and brain extracellular fluid, preventing diverse external stimuli from reaching neural tissue.^117^ Recent research has begun to clarify the roles of peripheral and central inflammation in depression, particularly in relation to the disruption of the BBB and its impact on depressive-like behaviors. This disruption allows peripheral signals to access the brain. Although a compromised BBB in MDD patients was noted 40 years ago,^118^ recent murine studies link chronic social stress to depressive behaviors through BBB disruption. The mechanism involves reduced Claudin-5 (Cldn5) expression, which permits peripheral IL-6 entry into the brain.^119^

PM_2.5_, due to its unique properties, can cross the BBB directly or via the nasal olfactory pathway.^120^^,^^121^ Additionally, matrix metalloproteinases (MMPs) degrade extracellular matrix proteins, which form the foundational structure of blood vessels.^122^ Breathing in air pollutants from traffic can enhance MMP activity and break down tight junction proteins in the brain’s blood vessels, leading to changes in BBB permeability and increased expression of neuroinflammatory markers.^123^ Male rats exposed to traffic-related air pollution (TRAP) at 5 months showed CA1-specific BBB damage: 75% less ZO-1 and twice the iron deposits (suggesting microhemorrhages).^124^ Heavy metals, such as cadmium and lead, significantly contribute to PM_2.5_-induced BBB leakage. Cadmium, a toxic pollutant, is associated with neurodegenerative diseases through BBB dysfunction. *In vitro* studies show that cadmium increases ROS levels and reduces the expression of proteins essential for inter-endothelial junctional integrity, like ZO-1, F-actin, and vimentin.^125^

### Changes of neural connectivity

Recent research has found that exposure to ambient air pollutants at ages 9–10 is associated with distinct changes in network connectivity over two years, indicating potential long-term impacts on brain network maturation.^8^^,^^126^ An individual’s depression polygenic risk modulates how PM_2.5_ alters working memory and stress-information flow in brain networks. PM_2.5_ exposure interacts with genetic risk for depression to disrupt prefrontal-parietal brain networks involved in working memory and stress processing. Using dynamic causal modeling, Li et al. found that individuals with higher polygenic risk and elevated PM_2.5_ exposure exhibited amplified stress-related connectivity changes between the posterior inferior parietal cortex and dorsolateral prefrontal cortex during working memory manipulation tasks. These disruptions were most pronounced under social stress, correlating with slower reaction times and impaired reasoning. Spatial co-expression of depression genes (RBFOX1, ENOX1) mapped onto stress-connectivity changes solely among high-risk/high-exposure subjects. This suggests PM_2.5_ exacerbates dysfunction in genetically vulnerable prefrontal-parietal circuits, potentially through neuroinflammatory pathways.^127^

### Neuronal atrophy

In patients with depression and animal models with chronic stress, the dendritic branches and synaptic density of glutamatergic neurons in the PFC and hippocampus are significantly reduced, leading to a reduction in brain volume.^128^^,^^129^ PM_2.5_ exposure may exert multifaceted detrimental effects on neurons, ranging from structural damage and synaptic dysfunction to neuroinflammation and genetic dysregulation. PM_2.5_-induced neuronal atrophy, characterized by apoptosis, necrosis, reduced neuronal diameter, and decreased presynaptic vesicle density,^33^^,^^130^ may contribute to depression through multiple interconnected neurobiological pathways. Additionally, PM_2.5_ disrupts dendritic morphology and affects the myelin sheath, resulting in shortened dendritic length and diminished spine density, which may impair synaptic connectivity and neuronal communication.^131^ The structural changes induced by PM_2.5_ are correlated with reduced expression of synaptic proteins, such as PSD-95 and synaptophysin, which are essential for synaptic integrity and neurotransmitter release.^132^ Notably, PM_2.5_ exposure alters neurotransmitter dynamics, with elevated glutamate levels observed, while other neurotransmitters like glycine and γ-aminobutyric acid (GABA) remain largely unaffected.^4^^,^^133^ PM_2.5_ (particularly its organic components, such as PAH) persistently suppressed the expression of the key transcription factor Hoxa5 in male offspring, leading to dysregulation of genes critical for axonal growth and dendritic complexity, ultimately causing abnormal neural network development.^73^ In human brain models, PM_2.5_ penetrates the BBB, accumulates in brain tissue, and triggers astrogliosis, neuronal loss, and microglial infiltration. Under PM_2.5_ exposure, reactive astrocytes and neurons release interleukin-1β (IL-1β) and interferon-γ (IFN-γ), polarizing infiltrating microglia toward a proinflammatory M1 phenotype.^134^ These activated M1 microglia further exacerbate neuronal damage by releasing proinflammatory mediators and nitric oxide, leading to synaptic impairment, phospho-tau accumulation, and neuronal death.^134^ Elevated PM_2.5_ levels were significantly linked to increased serum levels of neural damage markers, including brain-derived neurotrophic factor (BDNF), neurofilament light (NfL), and protein gene product 9.5 (PGP9.5).^135^ The glutathione S-transferase theta 1 (GSTT1) gene plays a role in detoxifying harmful chemicals and combating oxidative stress.^136^ Neuron-specific enolase (NSE) is a protein found in brain cells that leaks into the bloodstream when these cells are damaged.^137^ Research indicates that even short-duration exposure to PM_2.5_ may harm brain cells, as evidenced by significantly increased NSE biomarker levels. Surprisingly, individuals with the GSTT1 gene showed a stronger link between PM_2.5_ exposure and elevated NSE levels compared to those without the gene.^135^ This is unexpected because the GSTT1 gene typically aids in detoxification, suggesting it might interact with PM_2.5_ in ways that trigger harmful pathways like inflammation. Finally, exposure to TRAP-induced age-, genotype-, and sex-dependent neuronal loss in rats, alongside enhanced microglial activation.^138^

### Neuroinflammation

Neuroinflammation is a core pathological process that plays a central mechanistic role in the development and progression of depression. NLRP3 activation increases IL-1β and IL-18, reducing BDNF levels and inducing depressive behaviors.^139^ MDD is associated with altered levels of proinflammatory cytokines, oxidative stress, and reduced neurotrophic factors.

Microglial activation was observed in both people and animals with depression. Clinical evidence shows that the number of activated microglia and the level of a microglial activation marker called translocator protein increase in the anterior cingulate cortex and prefrontal cortex in depression patients.^140^^,^^141^ PM_2.5_ exposure triggers microglial activation, promotes a proinflammatory phenotype, and induces oxidative stress, leading to neuroinflammation and neurotoxicity.^33^ The microglial activation plays a dual role in both protective and detrimental responses to PM_2.5_-induced neurotoxicity. Under PM_2.5_ exposure, microglia undergo proliferation and marked activation, characterized by an increase in cell numbers and morphological changes such as enlarged cell bodies and increased process complexity. This activation likely represents an inflammatory response aimed at clearing damaged neurons and synaptic debris.^142^ PM_2.5_ triggers microglial activation, promoting M1 polarization and disease-associated microglia (DAM) signatures through elevated inflammatory mediators (IL-1β, IL-6, TNFα, iNOS, TREM2, TLR2/4, COX-2) and diminished anti-inflammatory factors (IL-10, arginase-1).^143^ PM_2.5_ triggers oxidative stress (increased nitric oxide and ROS) and modulates signaling pathways, such as enhancing c-Jun N-terminal kinases (JNKs) phosphorylation and reducing Akt activation, leading to neuroinflammatory responses.^143^ Exposure to PM_2.5_, specifically nanosized particulate matter (nPM), induces microglial activation and promotes neuroinflammatory responses in microglia, including oxidative stress markers such as NO and lipid peroxidation, as demonstrated in BV-2 microglial cell models.^33^ However, the neurotoxic effects of nPM may vary significantly between batches due to differences in chemical composition, influencing the magnitude of inflammatory and oxidative responses. While *in vitro* assays in BV-2 cells, like NO induction, reflect batch-dependent toxicity, these responses did not directly correlate with *in vivo* outcomes, such as cortical Aβ40/42 accumulation or systemic inflammation.^144^ A central mechanism involves the upregulation of CCR5 in microglia, which drives their pro-inflammatory polarization and activates the TLR4-NF-κB neuroinflammation signaling.^30^ This inflammasome-driven pathway is further implicated in PM_2.5_-aggravated neuronal damage under amyloid-beta (Aβ) pathology, where microglial IL-1β release, dependent on NLRP3 activation, intensifies inflammation and neuronal injury in neuron-microglia co-culture.^145^ These effects correlate with BACE1 upregulation, mediated by NF-κB p65-induced suppression of miRNA-574-5p, a microRNA targeting BACE1.^146^ Collectively, these studies demonstrate that PM_2.5_ exposure drives neuroinflammation and depressive pathology through microglial activation and NLRP3 inflammasome-mediated cytokine release.

Astrocytes play a key role in producing cytokines, contributing to depressive pathogenesis. Astrocytes protect neurons from oxidative stress by producing glutathione and activating Nrf2, a key antioxidant regulator.^147^ The hippocampus and PFC both exhibit volume reduction in depression, which is associated with decreased astrocyte density and neuronal synaptic loss.^148^^,^^149^ Patients with depression have chronic neuroinflammation in their brains, involving the activation of astrocytes.^150^ The contribution of astrocytes to depression pathogenesis arises through mechanisms such as structural and functional degeneration, dysregulated HPA axis activity, and neuroinflammation.^31^ Stress triggers astrocytes to initiate inflammatory responses via the NLRP3 inflammasome, leading to pyroptosis and irregular ATP release, which worsen neuroinflammation.^151^ Exposure to PM_2.5_ during prenatal development activates astrocytes in adult animals, as evidenced by elevated GFAP immunoreactivity, a marker of astrocyte activation, and increased COX2 protein levels in the brain, indicative of sustained neuroinflammatory responses.^152^ These astrocytic changes are associated with increased expression of Arg1 (an anti-inflammatory marker), suggesting PM_2.5_ promotes a pro-inflammatory astrocyte phenotype.^152^ Furthermore, PM_2.5_-induced cytokine dysregulation in the brain and spleen implies systemic immune-brain interactions, with astrocytes potentially contributing to chronic neuroinflammation.^152^ PM_2.5_ exposure activates astrocytes by triggering the NF-κB signaling pathway,^153^ which drives neuroinflammation, oxidative stress, and hypothalamic nerve injury, particularly under conditions of Nrf2 deficiency.^153^ Beyond neural impacts, prolonged PM_2.5_ inhalation triggers hypertension via sympathetic overactivation—potentially initiated by hypothalamic inflammation.^154^

### Oxidative stress

PM_2.5_, laden with toxic metals and organic compounds, triggers excessive ROS production, directly damaging cellular lipids, proteins, and DNA. Critically, PM_2.5_ exposure disrupts antioxidant defenses by suppressing key enzymes like superoxide dismutase and glutathione peroxidase, creating an oxidative imbalance that overwhelms cellular repair systems. This imbalance is particularly detrimental in the brain, where ROS accumulation promotes neuronal damage and apoptosis.^155^ Experimental evidence highlights the hypothalamus as a vulnerable target. Intranasal PM_2.5_ exposure in rats for four weeks selectively upregulated endoplasmic reticulum (ER) stress markers (CHOP, eIF2α, GRP78, P16) and NOX4, a major ROS-generating enzyme, in this region, while effects in the striatum, cortex, and hippocampus were less pronounced.^156^ The ER stress response, activated by unresolved protein misfolding, synergizes with NOX4-driven ROS to amplify cellular dysfunction. Notably, NO serves as a critical initiator in this cascade, linking PM_2.5_-induced oxidative stress to downstream inflammation and vascular impairment.^157^

### Other molecular pathways contributing to PM2.5-induced depression

Beyond the aforementioned molecular mechanisms of neuroinflammation, PM_2.5_ exposure also perturbs depression pathogenesis through intricate molecular pathways that span neuroendocrine, neurochemical, and metabolic domains. These pathways, which involve HPA axis dysfunction, neurotransmitter dysregulation, genetic and epigenetic aberrations, and alterations in the kynurenine pathway, collectively reshape the brain’s molecular landscape, precipitating depressive phenotypes. The HPA axis, as a key stress-responsive system, is highly susceptible to PM_2.5_-induced inflammatory signaling, leading to glucocorticoid dysregulation and hypothalamic neuroinjury.^158^ Concurrently, PM_2.5_ disrupts monoamine and glutamatergic neurotransmission, exacerbating synaptic dysfunction.^133^^,^^159^ At the genomic level, PM_2.5_ triggers epigenetic reprogramming and gene expression abnormalities that compromise neuroplasticity and stress resilience.^160^ Furthermore, perturbations in the kynurenine pathway shift tryptophan metabolism toward neurotoxic metabolites, amplifying inflammatory sequelae.^161^ These multifaceted molecular disruptions synergize to cause depression, underscoring the complexity of PM_2.5_’s neuropsychiatric impact.

#### HPA axis dysfunction

Stressful life events frequently act as triggers for depressive episodes, with the HPA axis playing a pivotal role in this process. Stress activates the HPA axis, resulting in the increased production of corticotropin-releasing hormone and the subsequent release of glucocorticoids such as cortisol.^162^ Prolonged elevation of glucocorticoids can damage neurons and contribute to depressive symptoms. Glucocorticoids also provide negative feedback to the HPA axis to maintain balance. Disruption of this feedback mechanism, coupled with chronically high glucocorticoid levels, is strongly associated with depression.^22^^,^^163^

PM_2.5_ exposure disrupts HPA axis regulation, likely through inflammatory pathways. PM_2.5_-triggered systemic inflammation elevates cytokines, which directly activate the HPA axis to provoke hypothalamic release of corticotropin-releasing hormone (CRH) and arginine vasopressin.^164^^,^^165^ Adrenocorticotropic hormone (ACTH), released from the pituitary in response to these signals, increases adrenal synthesis of cortisol (human) or corticosterone (rodent). Clinical evidence supports this mechanism: a recent longitudinal study using linear mixed-effects models revealed that PM_2.5_ exposure significantly elevates serum ACTH and cortisol levels in humans, implicating chronic HPA axis activation.^10^ Critically, the HPA axis’s responsiveness to PM_2.5_ highlights its role as a bridge between environmental pollutants and systemic physiological disruption, with cytokines serving as key mediators. Further research is needed to dissect whether PM_2.5_ components preferentially drive specific cytokine-HPA interactions, potentially informing targeted interventions.

#### Neurotransmitter

Depression is linked to deficiencies in monoamine neurotransmitters (serotonin/5-HT, dopamine/DA, norepinephrine/NE) and dysregulation of glutamate and ATP.^22^ Emerging evidence highlights that PM_2.5_ disrupts neurotransmitter homeostasis, though its effects vary significantly depending on exposure dose, duration, and the inherent sensitivity of specific neurotransmitter systems. In acute high-dose exposure models, PM_2.5_ induces pronounced neurotransmitter imbalances. For instance, metabolomics analysis suggested that whole-body inhalation of PM_2.5_ at 400 μg/m^3^ in mice activated the dopamine pathway while suppressing glycine metabolism, indicating pathway-specific hyperactivities.^166^ Similarly, tracheal perfusion with PM_2.5_ at 20 mg/kg in rats selectively elevated glutamate levels without altering serine, glycine, or γ- GABA, suggesting glutamate excitotoxicity as a high-dose response.^133^ In contrast, chronic exposure to lower PM_2.5_ concentrations elicits subtler perturbations. Mice exposed to 48 μg/m^3^ PM_2.5_ for three months showed only mild reductions in norepinephrine and the serotonin metabolite 5-hydroxyindoleacetic acid (5-HIAA), with no significant changes in serotonin (5-HT), dopamine, or levodopa levels.^25^ These findings collectively suggest that PM_2.5_ exposure could induce significant alterations in neurotransmitter systems, with the specific effects being highly dependent on exposure parameters and the susceptibility of different neurotransmitter pathways.

#### Genetic and epigenetic anomaly

Genetics accounts for 30%–50% of depression risk, highlighting its major role in the disorder’s development.^167^ Genome-wide association studies have mapped over 100 genetic loci conferring increased susceptibility to depression, underscoring its genetic complexity. These include genes involved in presynaptic vesicle trafficking, dopaminergic neurotransmission, glutamate signaling, and neuronal calcium signaling.^168^^,^^169^^,^^170^ DNA methylation, histone modifications (including acetylation/deacetylation), and non-coding RNAs constitute major epigenetic pathways. Acetylation of histones generally activates genes, while deacetylation suppresses them. In individuals with depression, patterns of histone modification are altered, particularly in brain regions associated with stress response, such as the hippocampus and prefrontal cortex.^171^

PM_2.5_ exposure drives profound alterations in gene expression through epigenetic reprogramming and direct transcriptional dysregulation, contributing to neurodevelopmental and neurodegenerative outcomes.^172^ Key epigenetic changes include hypermethylation of DNA repair genes (APEX1 and ERCC4) and hypomethylation of apoptosis-related genes (DAPK1),^173^^,^^174^ skewing cellular responses toward genomic instability and programmed cell death in depression.^175^^,^^176^^,^^177^ These effects are compounded by PM_2.5_-induced upregulation of DNA methyltransferase-1 (DNMT1), which represses neuroplasticity-associated genes like RELN, critical for synaptic maturation and neuronal migration.^142^ Exposure to PM_2.5_ in rats induces hypermethylation of the SHANK3 gene, which suppresses the expression of the SHANK protein. This downregulation disrupts SHANK3-mediated synaptic protein interactions, ultimately impairing synaptic function.^178^ In people genetically predisposed to depression, it disrupts brain networks involved in stress, which suggests that PM_2.5_ may activate depression risks in vulnerable individuals.^127^ GSTT1 is an enzyme that aids detoxification by binding glutathione to toxic compounds. Surprisingly, individuals born without this enzyme experience reduced neurological harm from PM_2.5_ air pollution exposure.^135^ Notably, the impacts of PM_2.5_ extend to developmental windows. Maternal exposure reduces placental expression of neurodevelopmental genes, including BDNF and synapsin-1, and induces leptin promoter methylation in offspring.^179^^,^^180^

#### Kynurenine pathway

The kynurenine pathway is the primary route for tryptophan metabolism, with approximately 95%–99% of tryptophan being metabolized through this pathway.^181^ Initially, tryptophan is converted into kynurenine, which is further metabolized by various enzymes into several neuroactive metabolites, including kynurenic acid, which has neuroprotective properties, and quinolinic acid (QUIN), which is neurotoxic. Kynurenic acid exerts its neuroprotective effects by antagonizing N-methyl-D-aspartate (NMDA) receptors, whereas QUIN increases excitotoxicity in glutamatergic neurons by activating NMDA receptors, leading to neurodegeneration.^182^^,^^183^ In the context of depression, peripheral evidence indicates an imbalance in kynurenine pathway metabolites in patients, characterized by decreased levels of kynurenic acid and increased activity of QUIN, potentially enhancing neurotoxic effects and contributing to the pathophysiology of depression.^184^ However, central nervous system studies reveal that depressed patients exhibit decreased kynurenic acid and kynurenine levels and increased QUIN levels.^185^

Emerging evidence suggests that chronic PM_2.5_/PM_10_ exposure perturbs the kynurenine pathway. In a cross-sectional study analyzing blood metabolites via 1H-NMR, individuals with long-term high PM exposure dysregulated the kynurenine pathway compared to low-exposure controls. Strikingly, high PM exposure elevated nearly all measured tryptophan metabolites—including serotonin, kynurenic acid, and picolinic acid—while paradoxically suppressing QUIN and nicotinamide adenine dinucleotide (NAD^+^), key products of the pathway’s neuroactive branch. Enzymatic shifts further underscored this imbalance: decreased indoleamine 2,3-dioxygenase (IDO) and formamidase activities contrasted with increased kynureninase and kynurenine monooxygenase, suggesting PM_2.5_ may redirect tryptophan metabolism toward pro-inflammatory intermediates and away from neuroprotective endpoints like NAD^+,^^161^ converging to drive depressive pathophysiology. However, another study shows that BALB/c mice exposed to 12 weeks of PM_2.5_ at 500 μg/m^3^ showed reduced kynurenic acid and elevated QUIN levels, aligning with the proposed mechanism that PM_2.5_ disrupts the kynurenine pathway, potentially contributing to depression by reducing neuroprotective kynurenic acid and increasing neurotoxic QUIN, which enhances neuronal excitotoxicity.^186^

Notably, these two studies report apparently opposite patterns regarding quinolinic acid. In the human cross-sectional study by Jaikang et al., long-term high PM exposure was associated with lower circulating QUIN and NAD^+^ levels, despite elevations in several upstream metabolites.^161^ In contrast, Park et al. observed higher QUIN and lower kynurenic acid in mice chronically exposed to PM_2.5_, consistent with a shift toward the neurotoxic branch of the kynurenine pathway. This discrepancy suggests that PM_2.5_-induced alterations in tryptophan metabolism may be highly context-dependent.^186^

Several factors may account for these divergent findings. First, the studies differ in species and experimental context: Jaikang et al. examined chronically exposed human adults living in polluted environments, whereas Park et al. used BALB/c mice subjected to 12 weeks of controlled PM_2.5_ exposure at 500 μg/m^3^, a relatively high concentration that may elicit more overt neuroinflammatory responses. Second, the exposure pattern and timing of assessment are likely to differ. Human study captures steady-state blood metabolites under long-term, real-world exposure with multiple potential modifiers (diet, comorbidities, medications, genetics), whereas the animal study assesses a specific post-exposure time point in healthy mice, which may reflect an earlier or more acute phase of pathway activation. Third, the biofluid matrix and analytical platforms (^1^H-NMR in humans vs. targeted assays in mice) can influence the relative detection of individual metabolites. Finally, chronic high exposure in humans may induce compensatory or feedback mechanisms that downregulate the QUIN/NAD^+^ branch over time, whereas chronic exposure in mice might preferentially reveal the initial shift toward neurotoxic metabolite production. Taken together, these considerations suggest that PM_2.5_ may perturb the kynurenine pathway in a dynamic, phase- and species-specific manner, and that the balance between neuroprotective (kynurenic acid) and neurotoxic (QUIN) metabolites may vary across different stages of exposure and disease progression.

Overall, the available data are too limited and heterogeneous to define a single, consistent pattern of kynurenine pathway dysregulation in response to PM_2.5_. Rather than a uniform increase or decrease in QUIN, current findings point to complex, context-dependent remodeling of tryptophan metabolism. Future longitudinal and mechanistic studies that ideally integrate brain and peripheral measures across different exposure windows are needed to clarify how PM_2.5_-driven alterations of the kynurenine pathway contribute to depression risk.

### Indirect mechanisms: Peripheral barrier dysfunction and peripheral-to-central communication

In real-world exposure scenarios, only a small fraction of inhaled PM_2_._5_ is expected to reach the brain directly via olfactory or vascular routes. Instead, most particles initially interact with peripheral barrier systems that constitute the primary interfaces with the external environment. These include the respiratory mucosa, gastrointestinal epithelium, skin, and ocular surface. Accumulating evidence indicates that PM_2_._5_-induced disruption of these barriers can elicit chronic local and systemic inflammation, immune dysregulation, metabolic disturbances, and neuroendocrine imbalance, which may indirectly influence brain function and increase vulnerability to depression through lung-brain, gut-brain, and skin-brain communication pathways.

Respiratory mucosa and the lung-brain axis: the conducting airways and alveolar surface are the principal deposition sites for inhaled PM_2_._5_. Epidemiological studies in children show that long- and short-term exposure to specific PM_2_._5_ components, particularly black carbon, organic carbon, ammonium, and sulfate, is associated with higher asthma incidence and reduced lung function, highlighting a sustained burden of airway inflammation and impaired respiratory capacity.^187^ Experimental work in mice demonstrates that whole-body PM_2_._5_ exposure induces inflammatory cell infiltration and structural alterations of the lung epithelium, with prominent involvement of macrophages and neutrophils.^188^ While alveolar type II (AT2) cells mount a rapid regenerative response to repair the gas-exchange barrier, the reparative capacity of airway progenitors such as club cells is compromised, potentially due to oxidant production by recruited immune cells.^188^ At the cellular level, PM_2_._5_ exposure alters the mechanical properties of primary AT2 cells, increasing membrane deformability and permeability, and impairs their proliferative and self-renewal potential while inducing senescence markers (p53, γ-H2A.X, P16^ink4a^, P21).^189^ These findings are consistent with a broader literature indicating that airway and alveolar epithelial stem cells are key targets of airborne toxicants and that their regenerative dysfunction can compromise mucosal integrity and repair.^190^

Single-cell RNA-sequencing further reveals cell-type-specific transcriptomic reprogramming in juvenile lungs after PM_2_._5_ exposure. Alveolar macrophages show upregulated oxidative phosphorylation and downregulated antibacterial defense pathways; CD209^+^ dendritic cells exhibit impaired antigen presentation and altered energy metabolism; and ciliated epithelial cells activate interferon signaling, while fibroblasts increase ribosomal protein translation and calcium channel regulation.^191^ PM_2_._5_ also reshapes intercellular communication networks between immune and structural cells,^191^ suggesting a sustained remodeling of pulmonary immune homeostasis. Collectively, these alterations in lung barrier integrity, immune cell activation, and regenerative capacity are likely to increase systemic release of pro-inflammatory cytokines and reactive species, chronically stimulate the HPA axis, and engage lung-brain neural pathways (vagal afferents), thereby indirectly modulating stress and mood-regulatory circuits in the brain.

Intestinal barrier and the gut-brain axis: the gastrointestinal tract is another major route by which PM_2_._5_ can enter the body, either through mucociliary clearance of inhaled particles or ingestion of contaminated food and water. *In vitro* models using human Caco-2 intestinal epithelial monolayers show that PM_2_._5_ exposure reduces transepithelial electrical resistance and increases paracellular flux of FITC-dextran, indicating compromised barrier integrity.^192^ These functional changes are accompanied by downregulation of β-catenin/CTNNB1, increased expression of TNF-α and IL-6, elevated ROS and MDA, and activation of PI3K/AKT signaling; pharmacological inhibition of PI3K or scavenging of ROS partially restores β-catenin expression and alleviates hyperpermeability.^192^ *In vivo*, chronic oropharyngeal exposure to ambient PM_2_._5_ leads to increased colonic epithelial proliferation, shortened colon length, and gut microbiota dysbiosis resembling inflammatory bowel disease.^193^ At the cellular level, PM_2_._5_ and standard reference material induce autophagy dysregulation, lysosomal membrane damage, and IL-8 production in human colon cells, with circulating IL-8 levels correlating with anthropogenic PM_2_._5_ indicators in exposed populations.^193^

Subchronic exposure to concentrated ambient PM_2_._5_ for two months in mice causes simultaneous lung and intestinal injury, systemic inflammatory responses, and widespread alterations in serum and bronchoalveolar lavage fluid metabolites, including glutamate, glutamine, formate, pyruvate, and lactate.^194^ Notably, both gut and lung microbiota exhibit reduced richness and compositional shifts that strongly associate with these metabolic abnormalities,^194^ underscoring the role of the gut-lung–metabolism axis in PM_2_._5_ toxicity. Given the well-established links between increased intestinal permeability (“leaky gut”), microbiota dysbiosis, systemic inflammation, and depression, PM_2_._5_-induced disruption of the intestinal barrier is a plausible indirect pathway contributing to mood disorders via immune activation, microbial metabolite signaling (short-chain fatty acids, tryptophan derivatives), and HPA axis dysregulation.

Skin barrier, autonomic reactivity, and neuroendocrine signaling: The skin, as the largest organ and a major environmental interface, is continuously exposed to airborne PM_2_._5_. Large birth-cohort analyses indicate that higher prenatal and early postnatal PM_2_._5_ exposure is associated with increased risk of atopic dermatitis in childhood, with particularly strong effects during late gestation and early life and a steep risk increase above ∼65 μg/m^3.^^195^ Atopic dermatitis is characterized by epidermal barrier dysfunction and chronic Th2-driven inflammation and is itself associated with elevated rates of depression and anxiety. At the cellular level, PM_2_._5_-exposed sebocytes show transcriptional alterations in xenobiotic and lipid metabolism, inflammation, oxidative stress, and barrier-related pathways; experimentally, PM_2_._5_ increases lipid synthesis and peroxidation, upregulates inflammatory cytokines, and disrupts factors critical for cell barrier function, while also altering steroid hormone biosynthesis and retinol metabolism.^196^ These findings suggest that PM_2_._5_ can perturb the cutaneous immune-endocrine milieu, with potential systemic consequences.

From a physiological stress perspective, adolescents living in neighborhoods with higher PM_2_._5_ concentrations exhibit greater autonomic reactivity to social-evaluative stress, as indexed by reduced heart rate variability and elevated skin conductance during a trier social stress test, independent of socioeconomic factors.^197^ Importantly, the association between PM_2_._5_ and heightened autonomic response is strongest among youths reporting more severe anxiety and depressive symptoms.^197^ These data imply that PM_2_._5_ may sensitize stress-response systems, including the autonomic nervous system and, by extension, the HPA axis, in psychologically vulnerable individuals. Chronic cutaneous barrier dysfunction, pruritus-induced sleep disturbance, and low-grade systemic inflammation arising from PM_2_._5_-related skin pathology may therefore act in concert to increase susceptibility to depression.

Ocular surface as an exposed mucosa and potential eye-brain communication: The ocular surface is directly exposed to atmospheric PM and, unlike many other organs, is difficult to shield from ambient air. A comprehensive review of *in vitro*, *in vivo*, and epidemiologic data concludes that PM_10_ and PM_2_._5_ induce inflammatory responses, oxidative stress, DNA damage, mitochondrial impairment, and impaired proliferation and migration of ocular surface cells, leading to defective wound healing, altered tear composition, and increased risk of dry eye disease, blepharitis, conjunctivitis, keratitis, limbal stem cell deficiency, and pterygium.^198^ Mechanistically, human corneal epithelial cells exposed to PM_2_._5_ show dose-dependent reductions in viability and activation of the NLRP3 inflammasome-mediated pyroptosis pathway, including upregulation of NLRP3, ASC, caspase-1, gasdermin D, IL-1β, and IL-18, accompanied by increased ROS formation; these changes are recapitulated in mouse corneas after chronic topical PM_2_._5_ application.^199^ Other studies reveal that PM_2_._5_ alters corneal biomechanical properties in humans and rodents and that intracellular plasminogen activator inhibitor 2 (PAI-2) modulates PM_2_._5_-induced autophagy and inflammation via a PAI-2/myosin II/F-actin/YAP positive feedback loop, with tear PAI-2 serving as a potential biomarker of early PM_2_._5_-related corneal damage.^200^ PM_2_._5_ also induces elevated intraocular pressure in mice through activation of the NLRP3/caspase-1/gasdermin D/IL-1β axis, which can be mitigated by κ-carrageenan treatment.^201^

Although direct links between ocular surface pathology and depression have not been extensively studied, chronic eye discomfort, pain, and visual impairment can substantially reduce quality of life, disrupt sleep, and contribute to psychological distress. Moreover, ocular surface inflammation may add to the overall systemic inflammatory burden. Together, these observations position the eye as an additional PM_2_._5_-exposed mucosal interface whose injury could indirectly influence brain function via humoral and neuroimmune pathways.

In summary, converging evidence indicates that PM_2_._5_-induced dysfunction of peripheral barrier systems, including respiratory mucosa, gut epithelium, skin, and ocular surface, generates chronic local and systemic inflammatory signals, alters microbiota composition and metabolic profiles, and perturbs autonomic and neuroendocrine regulation. These peripheral pathologies are well placed to influence central nervous system function through circulating cytokines, microbial metabolites, vagal and sensory afferents, and sustained HPA axis activation. While direct translocation of PM_2_._5_ into the brain via olfactory and vascular routes remains mechanistically important, these indirect peripheral-to-central pathways may represent the more frequent and clinically relevant routes by which environmental PM_2_._5_ exposure contributes to depression risk. Future longitudinal and mechanistic studies are needed to disentangle the relative contributions of direct versus indirect mechanisms and to clarify how specific barrier organ pathologies and comorbid conditions (asthma, atopic dermatitis, inflammatory bowel disease, ocular surface disease) interact with PM_2_._5_ exposure to shape vulnerability to depression.

### Integrated mechanistic model: Interacting pathways and vicious cycles

The preceding sections have deliberately examined individual pathways, including neuroinflammation, oxidative stress, HPA axis dysregulation, neurotransmitter imbalance, genetic and epigenetic changes, and kynurenine pathway alterations, in relative isolation. However, *in vivo*, these processes are highly interconnected and mutually reinforcing. Rather than acting as parallel, independent routes, they form a network of feed-forward loops that collectively drive the onset and maintenance of depressive pathology following PM_2_._5_ exposure.^11^^,^^142^^,^^150^

At the systems level, inhaled PM_2.5_ and its toxic constituents (transition/heavy metals, black carbon, PAHs, and other organics) first trigger peripheral inflammation and oxidative stress in the lungs, circulation, and gut.^9^^,^^142^^,^^155^ Pulmonary macrophage activation, endothelial dysfunction, and alterations of the lung and gut microbiota lead to elevated pro-inflammatory cytokines (IL-1β, IL-6, TNF-α) and excess ROS.^9^^,^^10^^,^^164^^,^^202^ These peripheral signals have at least three major consequences. First, they could promote BBB compromise through upregulation of matrix MMPs and downregulation of tight junction proteins (ZO-1, claudins), as well as metal-induced endothelial injury.^123^^,^^124^^,^^125^ Second, they propagate central neuroinflammation, as circulating cytokines and activated immune cells gain easier access to the brain parenchyma.^33^^,^^119^^,^^142^ Third, they chronically stimulate the HPA axis, increasing ACTH and cortisol/corticosterone levels.^10^^,^^158^^,^^165^

Once in the brain, PM_2_._5_ particles and associated metals further amplify microglial and astroglial activation, creating a self-sustaining neuroinflammatory milieu.^32^^,^^33^^,^^153^ Microglia adopt a pro-inflammatory phenotype (M1/DAM-like), producing IL-1β, IL-6, TNF-α, iNOS, and ROS, while astrocytes upregulate GFAP, COX-2, and NF-κB activity.^33^^,^^143^^,^^152^ These glial responses impair synaptic plasticity, reduce BDNF signaling, and promote neuronal apoptosis and dendritic spine loss in mood-regulating regions such as the hippocampus and prefrontal cortex.^128^^,^^129^^,^^149^ At the same time, PM_2_._5_-induced oxidative stress and mitochondrial dysfunction further increase ROS production, creating a vicious cycle between oxidative damage and neuroinflammation.^69^^,^^142^^,^^155^

HPA axis dysregulation both arises from and feeds back into these inflammatory processes.^22^^,^^150^^,^^162^ Pro-inflammatory cytokines stimulate hypothalamic CRH and vasopressin release, driving ACTH secretion and glucocorticoid overproduction.^162^^,^^164^^,^^165^ Chronic glucocorticoid excess, in turn, sensitizes microglia and peripheral immune cells, exacerbates oxidative stress, and impairs neurogenesis, particularly in the hippocampus.^128^^,^^150^^,^^163^ Thus, HPA overactivation constitutes a key feed-forward loop linking PM_2_._5_-induced immune activation to structural and functional brain changes characteristic of depression.^10^^,^^17^^,^^158^

Within this inflammatory and endocrine context, neurotransmitter systems and tryptophan metabolism are profoundly altered.^133^^,^^161^^,^^166^^,^^186^ PM_2_._5_ exposure can induce glutamatergic excitotoxicity, modestly reduce monoaminergic tone (serotonin and norepinephrine), and perturb dopaminergic signaling through phenyl-containing compounds that interfere with DRD1.^24^^,^^25^^,^^133^ In parallel, inflammatory signaling shifts the kynurenine pathway toward neuroactive metabolites.^181^^,^^184^^,^^185^ Chronic PM_2_._5_/PM_10_ exposure may remodel the kynurenine pathway in a context-dependent manner, reducing neuroprotective kynurenic acid and favoring accumulation of neurotoxic quinolinic acid in animal models, thereby enhancing NMDA receptor-mediated excitotoxicity.^161^^,^^183^^,^^186^ These changes in neurotransmission and tryptophan catabolism provide a mechanistic bridge from inflammation and oxidative stress to anhedonia, cognitive deficits, and negative affect.^22^^,^^184^^,^^185^

At the cellular and circuit levels, the convergence of neuroinflammation, oxidative stress, HPA dysregulation, and neurotransmitter imbalance culminates in synaptic dysfunction, neuronal atrophy, and large-scale network alterations.^107^^,^^128^^,^^142^ Reduced dendritic complexity and synaptic protein expression (PSD-95, synaptophysin) weaken connectivity within prefrontal-limbic circuits, while PM_2_._5_-related changes in functional connectivity of prefrontal-parietal networks are especially pronounced in genetically susceptible individuals.^127^^,^^129^^,^^132^ Over time, these micro- and macro-structural changes manifest as decreased volumes of the hippocampus, prefrontal cortex, and other emotion-processing regions, as documented in neuroimaging studies.^17^^,^^108^^,^^109^^,^^113^

Finally, genetic and epigenetic factors modulate vulnerability at multiple nodes of this network.^167^^,^^168^^,^^172^ PM_2_._5_-induced DNA methylation changes in genes related to DNA repair, apoptosis, synaptic function (RELN, SHANK3, BDNF), and antioxidant defense (NRF, GSTT1) may lower the threshold for developing depression under environmental stress.^135^^,^^136^^,^^142^^,^^173^^,^^174^^,^^175^^,^^177^^,^^178^^,^^180^ Individuals with high polygenic risk for depression or specific detoxification genotypes appear particularly sensitive to PM_2_._5_-related disruptions in brain connectivity and neural damage markers.^7^^,^^127^^,^^135^

**Figure 3:**
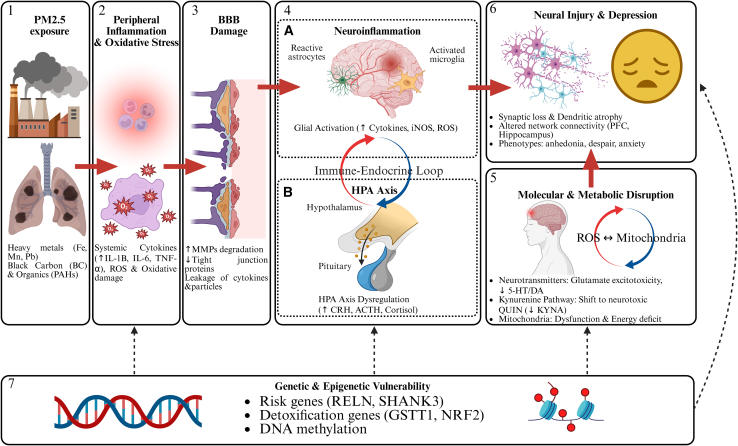
Integrated mechanistic model of PM_2.5_-induced depression: interacting pathways and vicious cycles Inhaled PM_2.5_ and its constituents induce peripheral inflammation and oxidative stress (lungs/gut), which disrupts the BBB. This allows the infiltration of cytokines and particles, triggering central neuroinflammation (microglial/astroglial activation) and chronic HPA axis dysregulation. These systems form a feedforward immune-endocrine loop (vicious cycle 1). The inflammatory milieu drives metabolic shifts, including mitochondrial dysfunction (creating an ROS feedback loop, vicious cycle 2) and the kynurenine pathway shift toward neurotoxic quinolinic acid (QUIN). Collectively, these interactions lead to neurotransmitter imbalance (glutamate excitotoxicity) and synaptic/neuronal atrophy in mood-regulating regions, culminating in depressive phenotypes. Genetic and epigenetic factors could act as modulators across these stages.

Taken together, current data support a unified model in which PM_2_._5_ acts as a systems-level stressor: initiating peripheral inflammation and oxidative stress, compromising the BBB, provoking central neuroinflammation, and chronically activating the HPA axis.^11^^,^^142^^,^^155^^,^^158^ These processes interact with one another and with neurotransmitter and kynurenine pathway disturbances to form interlocking vicious cycles that progressively damage stress- and mood-regulatory circuits.^107^^,^^150^^,^^184^^,^^185^ This integrated framework (Figure 3) underscores that PM_2_._5_-related pathways should not be viewed as isolated mechanisms, but as components of a dynamic, self-amplifying network that confers increased risk for MDD.^11^^,^^22^^,^^142^

Collectively, mechanistic studies indicate that PM_2.5_ perturbs brain function through a network of interacting pathways rather than a single linear cascade. The most robust evidence supports roles for neuroinflammation, oxidative stress, HPA-axis dysregulation, and synaptic and neuronal atrophy in mood-regulating circuits, with additional contributions from neurotransmitter imbalance, epigenetic reprogramming, alterations in the kynurenine pathway, and dysfunction of peripheral barrier organs. Strengths of this body of work include the use of diverse experimental models, convergent findings across molecular, cellular, circuit, and neuroimaging levels, and partial alignment with established pathophysiological models of depression. However, much of the mechanistic evidence derives from acute or subchronic exposures in rodents, selected strains and sexes, and reductionist *in vitro* systems, and human data remain largely indirect. Longitudinal human studies integrating refined exposure assessment with multimodal biomarkers and neuroimaging are needed to validate these pathways in humans and quantify their relative contributions. In the concluding section, we outline key research priorities and potential intervention targets that arise from this integrative mechanistic framework.

From a broader disease-mechanism perspective, many of the pathways implicated in PM_2.5_-related depression closely resemble those engaged by other environmental stressors and neurodegenerative disorders. Chronic psychological stress, for example, is well known to activate the HPA axis, induce sustained elevations of glucocorticoids, promote microglial and astroglial activation, and drive oxidative stress and synaptic loss in the hippocampus and prefrontal cortex, which are core changes that underpin stress-related major depression.^203^^,^^204^ Accumulated evidence suggests that PM_2.5_ exposure converges on these possible pathways via inflammatory and metabolic perturbations in the peripheral organs, including the lung, gut, skin, and ocular surface, which could indirectly induce central stress and immune responses.

In addition, there is substantial overlap between PM_2.5_-induced brain injury and early pathophysiological processes in neurodegenerative diseases such as Alzheimer’s disease. Both are characterized by chronic neuroinflammation, BBB breakdown, cerebrovascular dysfunction, mitochondrial impairment, and progressive synaptic and neuronal loss in memory- and mood-related regions. Certain PM_2.5_ constituents, including transition metals, sulfate-rich aerosols, and organic compounds such as PAHs, have been linked to enhanced amyloid-β aggregation, tau hyperphosphorylation, and α-synuclein pathology, providing a mechanistic bridge between air pollution, neurodegeneration, and depression in later life.^205^^,^^206^ At the same time, PM_2.5_ exposure is distinctive in that it typically occurs as a complex, multicomponent, low-dose, lifelong environmental stressor that additionally targets peripheral barrier organs and microbiota. Thus, PM_2.5_ may be an environmental factor that exacerbates the effects of chronic psychosocial stress and neurodegenerative processes, positioning air pollution as a potential fertilizer for brain aging and mood disorders.^207^

## Conclusions and perspectives

The growing body of evidence reviewed here suggests a link between PM_2.5_ exposure and depression risk, supported by epidemiological data, preclinical models, and mechanistic insights. Chronic or acute exposure to PM_2.5_ consistently correlates with elevated depression prevalence across diverse populations. Complementary animal studies confirm that chronic PM_2.5_ exposure can induce depression-like behaviors. Notably, neurotoxic components of PM_2.5_ act synergistically or independently to disrupt neural homeostasis. Mechanistically, PM_2.5_ impacts the brain through multiscale pathways: structural alterations in stress-related regions, cellular dysfunction, and molecular perturbations. Neuroinflammation is a key mechanism, as shown by many studies where PM_2.5_ exposure triggered microglial activation and increased proinflammatory cytokines. However, emerging evidence also supports other molecular mechanisms of PM_2.5_-induced depression, including the HPA axis dysfunction, neurotransmitter imbalance, genetic and epigenetic anomaly, and the kynurenine pathway dysfunction.

Nevertheless, critical knowledge gaps remain. These include the mechanistic complexities of PM_2.5_-induced depression, the precise characterization of exposure-effect dynamics, and the translation of neurotoxicological findings into clinical applications. Human studies often lack fundamental data on exposure timing, regional differences, dose-response relationships, and individual susceptibility factors. While animal models clarify causality, interspecies differences limit direct translation to humans. The relative contributions of specific PM_2.5_ components to neurotoxicity remain poorly defined, hindering targeted interventions. Most mechanistic studies focus on isolated pathways; a system-level understanding of how PM_2.5_ disrupts neural networks is still emerging. While current research has largely focused on understanding the mechanisms of PM_2.5_-induced depression, translating these insights into therapeutic strategies remains an urgent yet underexplored frontier.

Since currently available studies are limited to short durations of PM_2.5_ exposure, longitudinal cohorts with high-resolution exposure assessment are needed to jointly measure PM_2.5_ components, peripheral inflammatory and endocrine markers, and depression outcomes, enabling mediation analyses. Future studies should also focus on source- and component-resolved analyses quantifying dose-response relationships for metals, black carbon, sulfates/nitrates, and organic compounds in animal models. With mechanistic insights underlying PM_2.5_’s action still lacking, mechanistic work should move toward cell-type-resolved and multiomics approaches in both central, including the key brain regions involved in depression (prefrontal cortex, hippocampus, and amygdala), and peripheral organs, to map vulnerable cellular targets and pathways. Future studies should also consider sex, exposure time window during development, and genetic susceptibility to interpret interindividual variability in PM_2.5_-related depression risk.

## Limitations of study

It is important to emphasize that most human studies are observational designs, which can only demonstrate a statistical association between PM_2.5_ exposure and depression, but cannot completely rule out residual confounding and reverse causality. However, the results from different regions and study designs show consistency in direction. Animal experiments and mechanistic studies provide biological interpretability. The current evidence collectively supports the plausibility of PM_2.5_ as a potential environmental risk factor for depression. Beyond limitations of the primary literature, several constraints of the present review should be acknowledged. Animal studies demonstrating the specific action of PM_2.5_ components in triggering depression are still in their infancy. The association between specific PM_2.5_ components and depression is primarily presented based on the common cellular/molecular changes that are affected by PM_2.5_ components and depression. Future studies using animal models are needed to demonstrate causal relationships. Moreover, heterogeneous experimental conditions, including animal species, exposure paradigms, doses, and outcome measures, differ substantially across studies; more animal studies will be required to further demonstrate neurotoxicity with chronic exposure to ambient air pollutants in real-life situations.

### Method details

#### Literature search and scope

A narrative review was conducted to provide an integrative overview of epidemiological, experimental, and mechanistic evidence on the association between PM_2_._5_ exposure and depression, with a particular emphasis on component-specific neurotoxicity and neural pathways.

We identified relevant publications through non-systematic searches of PubMed and Google Scholar up to 2026 Jan using combinations of the following keywords: “PM_2.5_,” “air pollution,” “depression,” “major depressive disorder,” “anxiety,” “neurotoxicity,” “animal model,” “mechanism,” “hippocampus,” “neuroinflammation,” “HPA axis,” “kynurenine,” and “component.” “MDD” is used to refer to clinically diagnosed unipolar major depression. “Depression” is used more broadly to encompass both diagnosed MDD and clinically relevant depressive symptoms as assessed by rating scales in epidemiological studies.

We primarily included (1) epidemiological studies reporting associations between PM_2_._5_ (or its components) and depression or depressive symptoms; (2) animal studies evaluating depression-like behaviors or relevant neurobiological outcomes following PM_2_._5_ exposure; and (3) mechanistic studies examining neural, cellular, or molecular pathways plausibly linking PM_2_._5_ to depression-related phenotypes.

#### Eligibility and evidence selection

Studies were considered relevant if they addressed at least one of the following topics: associations between PM_2.5_ or PM_2.5_ components and depression or depressive symptoms in human populations; depression like behaviors or related neurobiological outcomes after PM_2.5_ exposure in animal models; neurotoxic effects of specific PM_2.5_ components; or cellular, molecular, structural, and circuit level mechanisms plausibly linking PM_2.5_ exposure to depression related outcomes.

Priority was given to studies that provided direct evidence on PM_2.5_ exposure and depression related outcomes, studies with clearly described exposure assessment or experimental exposure paradigms, and mechanistic studies addressing pathways relevant to depression biology. Reviews and meta-analyses were used to contextualize the field and identify additional relevant primary studies.

Because this article is a narrative review rather than a systematic review, study identification and selection were not conducted according to PRISMA guidelines, and no formal risk of bias assessment was performed.

#### Evidence extraction and synthesis

Evidence was narratively synthesized across four major domains: epidemiological evidence, animal model evidence, component-specific neurotoxicity, and neural mechanisms. For epidemiological studies, extracted information included population characteristics, geographic location, exposure timing, exposure duration, PM_2.5_ or component exposure metrics, depression related outcomes, and main findings. For animal studies, extracted information included animal species and strain, sex and age when available, exposure method, exposure duration, exposure concentration, behavioral tests, neurobiological endpoints, and main findings.

Mechanistic evidence was organized according to major biological pathways implicated in depression, including neuroinflammation, oxidative stress, blood brain barrier disruption, HPA axis dysregulation, neurotransmitter imbalance, mitochondrial dysfunction, kynurenine pathway alterations, epigenetic reprogramming, synaptic dysfunction, neuronal atrophy, and brain network changes. Particular attention was given to evidence connecting peripheral exposure routes, including lung brain, gut brain, skin brain, and ocular surface related pathways, with central nervous system changes.

#### Figure and table preparation

Tables were prepared to summarize human epidemiological evidence, animal model evidence, component-specific neurotoxicity, and neural mechanisms linking PM_2.5_ exposure to depression-related outcomes. Figures were prepared to illustrate PM_2.5_ composition, exposure routes, and integrated mechanistic pathways. Figures 1, 2, and 3 and the graphical abstract were created with BioRender.com, and publication and licensing rights have been confirmed.

### Quantification and statistical analysis

No original quantitative analysis, meta-analysis, or statistical modeling was performed in this narrative review. Quantitative estimates reported in the text and tables were extracted from the published studies cited in the References.

### Additional resources

No additional resources, websites, clinical trial registrations, or protocol repositories were generated for this review.

## Acknowledgments

This work was supported by the Theme-based Research Scheme (T24-508/22-N) of the Research Grants Council of Hong Kong. Figures 1, 2, and 3 and the graphical abstract were created with BioRender.com, and publication and licensing rights have been confirmed.

## Author contributions

Conceptualization, Z.M. and S.-y.Y.; investigation, Z.M.; writing – original draft, Z.M.; writing – review and editing, Z.M. and S.-y.Y.; visualization, Z.M.; supervision, S.-y.Y.; funding acquisition, S.-y.Y.

## Declaration of interests

The authors declare no competing interests.

## Declaration of generative AI and AI-assisted technologies in the writing process

During the preparation of this work, the authors used ChatGPT by OpenAI and DeepSeek to assist with improving the readability and language of the manuscript. After using these tools, the authors reviewed, edited, and verified the content as needed and take full responsibility for the content of the publication. No generative AI or AI-assisted tools were used to create or alter the graphical abstract or figures.
